# A Game-Theoretic Approach to Information-Flow Control via Protocol Composition

**DOI:** 10.3390/e20050382

**Published:** 2018-05-18

**Authors:** Mário S. Alvim, Konstantinos Chatzikokolakis, Yusuke Kawamoto, Catuscia Palamidessi

**Affiliations:** 1 Computer Science Department, Universidade Federal de Minas Gerais (UFMG), Belo Horizonte-MG 31270-110, Brazil; 2 École Polytechnique, 91128 Palaiseau, France; 3 Centre National de la Recherche Scientifique (CNRS), 91190 Gif-sur-Yvette, France; 4 National Institute of Advanced Industrial Science and Technology (AIST), Tsukuba 305-8560, Japan; 5 INRIA Saclay, 91120 Palaiseau, France

**Keywords:** information leakage, quantitative information flow, game theory, algebraic properties

## Abstract

In the inference attacks studied in Quantitative Information Flow (QIF), the attacker typically tries to interfere with the system in the attempt to increase its leakage of secret information. The defender, on the other hand, typically tries to decrease leakage by introducing some controlled noise. This noise introduction can be modeled as a type of protocol composition, i.e., a probabilistic choice among different protocols, and its effect on the amount of leakage depends heavily on whether or not this choice is visible to the attacker. In this work, we consider operators for modeling visible and hidden choice in protocol composition, and we study their algebraic properties. We then formalize the interplay between defender and attacker in a game-theoretic framework adapted to the specific issues of QIF, where the payoff is information leakage. We consider various kinds of leakage games, depending on whether players act simultaneously or sequentially, and on whether or not the choices of the defender are visible to the attacker. In the case of sequential games, the choice of the second player is generally a function of the choice of the first player, and his/her probabilistic choice can be either over the possible functions (mixed strategy) or it can be on the result of the function (behavioral strategy). We show that when the attacker moves first in a sequential game with a hidden choice, then behavioral strategies are more advantageous for the defender than mixed strategies. This contrasts with the standard game theory, where the two types of strategies are equivalent. Finally, we establish a hierarchy of these games in terms of their information leakage and provide methods for finding optimal strategies (at the points of equilibrium) for both attacker and defender in the various cases.

## 1. Introduction

A fundamental problem in computer security is the leakage of sensitive information due to the correlation of secret values with observables, i.e., any information accessible to the attacker, such as, for instance, the system’s outputs or execution time. The typical defense consists of reducing this correlation, which can be done in, essentially, two ways. The first, applicable when the correspondence secret-observable is deterministic, consists of coarsening the equivalence classes of secrets that give rise to the same observables. This can be achieved with post-processing, i.e., sequentially composing the original system with a program that removes information from observables. For example, a typical attack on encrypted web traffic consists of the analysis of the packets’ length, and a typical defense consists of padding extra bits so as to diminish the length variety [1].

The second kind of defense, on which we focus in this work, consists of adding controlled noise to the observables produced by the system. This can be usually seen as a composition of different protocols via probabilistic choice.

**Example** **1** (Differential privacy)**.** Consider a counting query f, namely a function that, applied to a dataset x, returns the number of individuals in x that satisfies a given property. A way to implement differential privacy [2] is to add geometrical noise to the result of f, so as to obtain a probability distribution P on integers of the form $P\left(z\right)=c\;e^{\left|z-f(x)\right|}$, where c is a normalization factor. The resulting mechanism can be interpreted as a probabilistic choice on protocols of the form f(x),f(x)+1,f(x)+2,…,f(x)−1,f(x)−2,…, where the probability assigned to f(x)+n and to f(x)−n decreases exponentially with n.

**Example** **2** (Dining cryptographers)**.** Consider two agents running the dining cryptographers protocol [3], which consists of tossing a fair binary coin and then declaring the exclusive or *⊕* of their secret value x and the result of the coin. The protocol can be thought of as the fair probabilistic choice of two protocols, one consisting simply of declaring x and the other declaring x⊕1.

Most of the work in the literature of Quantitative Information Flow (QIF) considers passive attacks, in which the attacker only observes the system. Notable exceptions are the works [4,5,6], which consider attackers who interact with and influence the system, possibly in an adaptive way, with the purpose of maximizing the leakage of information.

**Example** **3** (CRIME attack)**.** Compression Ratio Info-leak Made Easy (CRIME) [7] is a security exploit against secret web cookies over connections using the HTTPS and SPDY protocols and data compression. The idea is that the attacker can inject some content a into the communication of the secret x from the target site to the server. The server then compresses and encrypts the data, including both a and x, and sends back the result. By observing the length of the result, the attacker can then infer information about x. To mitigate the leakage, one possible defense would consist of transmitting, along with x, also an encryption method f selected randomly from a set F. Again, the resulting protocol can be seen as a composition, using probabilistic choice, of the protocols in the set F.

**Example** **4** (Timing side-channels)**.** Consider a password-checker, or any similar system in which the user authenticates himself/herself by entering a secret that is checked by the system. An adversary does not know the real secret, of course, but a timing side-channel could reveal the part (e.g., which bit) of the secret in which the adversary’s input fails. By repeating the process with different inputs, the adversary might be able to fully retrieve the secret. A possible counter measure is to make the side channel noisy, by randomizing the order in which the secret’s bits are checked against the user input. This example is studied in detail in Section 7.

In all examples above, the main use of the probabilistic choice is to obfuscate the relation between secrets and observables, thus reducing their correlation; and hence, the information leakage. To achieve this goal, it is essential that the attacker never comes to know the result of the choice. In the CRIME example, however, if *f* and *a* are chosen independently, then (in general) it is still better to choose *f* probabilistically, even if the attacker will come to know, afterwards, the choice of *f*. In fact, this is true also for the attacker: his/her best strategies (in general) are to choose *a* according to some probability distribution. Indeed, suppose that $F=\{f_{1},f_{2}\}$ are the defender’s choices and $A=\{a_{1},a_{2}\}$ are the attacker’s and that $f_{1}\left(\cdot ,a_{1}\right)$ leaks more than $f_{1}\left(\cdot ,a_{2}\right)$, while $f_{2}\left(\cdot ,a_{1}\right)$ leaks less than $f_{2}\left(\cdot ,a_{2}\right)$. This is a scenario like the matching pennies in game theory: if one player selects an action deterministically, the other player may exploit this choice and get an advantage. For each player, the optimal strategy is to play probabilistically, using a distribution that maximizes his/her own gain for all possible actions of the attacker. In zero-sum games, in which the gain of one player coincides with the loss of the other, the optimal pair of distributions always exists, and it is called the saddle point. It also coincides with the Nash equilibrium, which is defined as the point at which neither of the two players gets any advantage in changing his/her strategy unilaterally.

Motivated by these examples, this paper investigates the two kinds of choice, visible and hidden (to the attacker), in a game-theoretic setting. Looking at them as language operators, we study their algebraic properties, which will help reason about their behavior in games. We consider zero-sum games, in which the gain (for the attacker) is represented by the leakage. While for the visible choice, it is appropriate to use the “classic” game-theoretic framework, for the hidden choice, we need to adopt the more general framework of the information leakage games proposed in [6]. This happens because, in contrast with standard game theory, in games with hidden choice, the payoff of a mixed strategy is a convex function of the distribution on the defender’s pure actions, rather than simply the expected value of their utilities. We will consider both simultaneous games, in which each player chooses independently, and sequential games, in which one player chooses his/her action first. We aim at comparing all these situations and at identifying the precise advantage of the hidden choice over the visible one.

To measure leakage, we use the well-known information-theoretic model. A central notion in this model is that of entropy, but here, we use its converse, vulnerability, which represents the magnitude of the threat. In order to derive results as general as possible, we adopt the very comprehensive notion of vulnerability as any convex and continuous function, as used in [4] and [8]. This notion has been shown [8] to be, in a precise sense, the most general information measure w.r.t. a set of fundamental information-theoretic axioms. Our results, hence, apply to all information measures that respect such fundamental principles, including the widely-adopted measures of Bayes vulnerability (also known as min-vulnerability, also known as (the converse of) Bayes risk) [9,10], Shannon entropy [11], guessing entropy [12] and *g*-vulnerability [13].

The main contributions of this paper are:

- We present a general framework for reasoning about information leakage in a game-theoretic setting, extending the notion of information leakage games proposed in [6] to both simultaneous and sequential games, with either a hidden or visible choice.
- We present a rigorous compositional way, using visible and hidden choice operators, for representing attacker’s and defender’s actions in information leakage games. In particular, we study the algebraic properties of visible and hidden choice on channels and compare the two kinds of choice with respect to the capability of reducing leakage, in the presence of an adaptive attacker.
- We provide a taxonomy of the various scenarios (simultaneous and sequential) showing when randomization is necessary, for either attacker or defender, to achieve optimality. Although it is well known in information flow that the defender’s best strategy is usually randomized, only recently has it been shown that when defender and attacker act simultaneously, the attacker’s optimal strategy also requires randomization [6].
- We compare the vulnerability of the leakage games for these various scenarios and establish a hierarchy of leakage games based on the order between the value of the leakage in the Nash equilibrium. Furthermore, we show that when the attacker moves first in a sequential game with hidden choice, the behavioral strategies (where the defender chooses his/her probabilistic distribution after he/she has seen the choice of the attacker) are more advantageous for the defender than the mixed strategies (where the defender chooses the probabilistic distribution over his/her possible functional dependency on the choice of the attacker). This contrast with the standard game theory, where the two types of strategies are equivalent. Another difference is that in our attacker-first sequential games, there may not exist Nash equilibria with deterministic strategies for the defender (although the defender has full visibility of the attacker’s moves).
- We use our framework in a detailed case study of a password-checking protocol. A naive program, which checks the password bit by bit and stops when it finds a mismatch, is clearly very insecure, because it reveals at each attempt (via a timing side-channel) the maximum correct prefix. On the other hand, if we continue checking until the end of the string (time padding), the program becomes very inefficient. We show that, by using probabilistic choice instead, we can obtain a good trade-off between security and efficiency.

### Plan of the Paper

The remainder of the paper is organized as follows. In Section 2, we review some basic notions of game theory and quantitative information flow. In Section 3, we introduce our running example. In Section 4, we define the visible and hidden choice operators and demonstrate their algebraic properties. In Section 5, the core of the paper, we examine various scenarios for leakage games. In Section 6, we compare the vulnerability of the various leakage games and establish a hierarchy among those games. In Section 7, we show an application of our framework to a password checker. In Section 8, we discuss related work, and finally, in Section 9, we conclude.

A preliminary version of this paper appeared in [14]. One difference with respect to [14] is that in the present paper, we consider both behavioral and mixed strategies in the sequential games, while in [14], we only considered the latter. We also show that the two kinds of strategies are not equivalent in our context (Example 10: the optimal strategy profile yields a different payoff depending on whether the defender adopts mixed strategies or behavioral ones). In light of this difference, we provide new results that concern behavioral strategies, and in particular:

- Theorem 3, which concerns the defender’s behavioral strategies in the defender-first game with visible choice (Game II),
- the second half of Theorem 6, which deals with the adversary’s behavioral strategies in the attacker-first game with hidden choice (Game VI).

Furthermore, in this paper, we define formally all concepts and provide all the proofs. In particular, we provide a precise formulation of the comparison among games with visible/hidden choices (Propositions 4 and 5, Corollaries 3–5) in Section 6. Finally, in Section 7, we provide a new result, expressed by Theorem 7, regarding the optimal strategies for the defender in the presence of a uniform prior on passwords.

## 2. Preliminaries

In this section, we review some basic notions from game theory and quantitative information flow. We use the following notation: Given a set I, we denote by DI the set of all probability distributions over I. Given μ∈DI, its support $\left(\mu \right)\overset{\mathrm{def}}{=}\left\{i\in I:\mu \left(i\right)>0\right\}$ is the set of its elements with positive probability. We use i←μ to indicate that a value i∈I is sampled from a distribution μ on I.  A set $S\subseteq R^{n}$ is convex if $ts_{0}+\left(1-t\right)s_{1}\in S$ for all $s_{0},s_{1}\in S$ and t∈[0,1]. For such a set, a function f:S→R is convex if $f\left(ts_{0}+\left(1-t\right)s_{1}\right)\leq tf\left(s_{0}\right)+\left(1-t\right)f\left(s_{1}\right)$ for all $s_{0},s_{1}\in S,t\in \left[0,1\right]$, and concave if −f is convex.

### 2.1. Basic Concepts from Game Theory

#### 2.1.1. Two-Player Games

Two-player games are a model for reasoning about the behavior of two players. In a game, each player has at its disposal a set of actions that he/she can perform, and he/she obtains some gain or loss depending on the actions chosen by both players. Gains and losses are defined using a real-valued payoff function. Each player is assumed to be rational, i.e., his/her choice is driven by the attempt to maximize his/her own expected payoff. We also assume that the set of possible actions and the payoff functions of both players are common knowledge.

In this paper, we only consider finite games, in which the set of actions available to the players is finite, which are also zero-sum games, so the payoff of one player is the loss of the other. Next, we introduce an important distinction between simultaneous and sequential games. In the following, we will call the two players defender and attacker.

#### 2.1.2. Simultaneous Games

In a simultaneous game, each player chooses his/her action without knowing the action chosen by the other. The term “simultaneous” here does not mean that the players’ actions are chosen at the same time, but only that they are chosen independently. Formally, such a game is defined as a tuple (following the convention of security games, we set the first player to be the defender) $\left(D,A,u_{d},u_{a}\right)$, where D is a nonempty set of defender’s actions, A is a nonempty set of attacker’s actions, $u_{d}:D\times A\to R$ is the defender’s payoff function and $u_{a}:D\times A\to R$ is the attacker’s payoff function.

Each player may choose an action deterministically or probabilistically. A pure strategy of the defender (respectively attacker) is a deterministic choice of an action, i.e., an element d∈D (respectively a∈A). A pair (d,a) is called pure strategy profile, and $u_{d}\left(d,a\right)$, $u_{a}\left(d,a\right)$ represent the defender’s and the attacker’s payoffs, respectively. A mixed strategy of the defender (respectively attacker) is a probabilistic choice of an action, defined as a probability distribution δ∈DD (respectively α∈DA). A pair (δ,α) is called mixed strategy profile. The defender’s and the attacker’s expected payoff functions for mixed strategies are defined, respectively, as:

$$\begin{array}{cc} U_{d}\left(\delta ,\alpha \right) & \overset{\mathrm{def}}{=}\underset{\begin{array}{c} d\leftarrow \delta \\ a\leftarrow \alpha \end{array}}{E}u_{d}\left(d,a\right)=\sum_{\begin{array}{c} d\in D\\ a\in A \end{array}}\delta \left(d\right)\alpha \left(a\right)u_{d}\left(d,a\right)\mathrm{and}:\\ U_{a}\left(\delta ,\alpha \right) & \overset{\mathrm{def}}{=}\underset{\begin{array}{c} d\leftarrow \delta \\ a\leftarrow \alpha \end{array}}{E}u_{a}\left(d,a\right)=\sum_{\begin{array}{c} d\in D\\ a\in A \end{array}}\delta \left(d\right)\alpha \left(a\right)u_{a}\left(d,a\right). \end{array}$$

A defender’s mixed strategy δ∈DD is the best response to an attacker’s mixed strategy α∈DA if $U_{d}\left(\delta ,\alpha \right)=\max_{\delta^{\prime }\in DD}U_{d}\left(\delta^{\prime },\alpha \right)$. Symmetrically, α∈DA is the best response to δ∈DD if $U_{a}\left(\delta ,\alpha \right)\;=\;\max_{\alpha^{\prime }\in DA}U_{d}\left(\delta ,\alpha^{\prime }\right)$. A mixed-strategy Nash equilibrium is a profile $\left(\delta^{*},\alpha^{*}\right)$ such that $\delta^{*}$ is the best response to $\alpha^{*}$ and vice versa. This means that in a Nash equilibrium, no unilateral deviation by any single player provides better payoff to that player. If $\delta^{*}$ and $\alpha^{*}$ are point distributions concentrated on some $d^{*}\in D$ and $a^{*}\in A$, respectively, then $\left(\delta^{*},\alpha^{*}\right)$ is a pure-strategy Nash equilibrium and will be denoted by $\left(d^{*},a^{*}\right)$. While not all games have a pure strategy Nash equilibrium, every finite game has a mixed strategy Nash equilibrium.

#### 2.1.3. Sequential Games

In a sequential game, players may take turns in choosing their actions. In this paper, we only consider the case in which each player moves only once, in such a way that one of the players (the leader) chooses his/her action first, and commits to it, before the other player (the follower) makes his/her choice. The follower may have total knowledge of the choice made by the leader, or only partial. We refer to the two scenarios by the terms perfect and imperfect information, respectively. Another distinction is the kind of randomization used by the players, namely whether the follower chooses probabilistically his/her action after he/she knows (partially or totally) the move of the leader, or whether he/she chooses at the beginning of the game a probabilistic distribution on (deterministic) strategies that depend on the (partial or total) knowledge of the move of the leader. In the first case, the strategies are called behavioral, in the second case mixed.

We now give the precise definitions assuming that the leader is the defender. The definitions for the case in which the leader is the attacker are analogous.

A defender-first sequential game with perfect information is a tuple $\left(D,\;D\;\to \;A,\;u_{d},u_{a}\right)$ where D, A, $u_{d}$ and $u_{a}$ are defined as in simultaneous games: The choice of an action d∈D represents a pure strategy of the defender. As for the attacker, his/her choice a∈A depends functionally on the prior choice *d* of the defender, and for this reason, the pure strategies of the attacker are functions $s_{a}:D\;\to \;A$. As for the probabilistic strategies, those of the defender are defined as in simultaneous games: namely, they are distributions δ∈DD. On the other hand, the attacker’s probabilistic strategies can be defined in two different ways: In the behavioral case, an attacker’s probabilistic strategy is a function $\phi_{a}:D\to D\left(A\right)$. Namely, the attacker chooses a distribution on his/her actions after he/she sees the move of the defender. In the mixed case, an attacker’s probabilistic strategy is a probability distribution $\sigma_{a}\in D\left(D\;\to \;A\right)$. Namely, the attacker chooses a priori a distribution on pure strategies. The defender’s and the attacker’s expected payoff functions for mixed strategies are defined, respectively, as:

$$\begin{array}{cc} \mathrm{Behavioral}\;\mathrm{case}:\;\; & \left\{\begin{array}{ccccc} U_{d}\left(\delta ,\phi_{a}\right) & \overset{\mathrm{def}}{=} & \underset{d\leftarrow \delta }{E}\;\underset{a\leftarrow \phi_{a}\left(d\right)}{E}u_{d}\left(d,a\right) & = & \sum_{d\in D}\delta \left(d\right)\sum_{a\in A}\phi_{a}\left(d\right)\left(a\right)\;u_{d}\left(d,a\right)\\ U_{a}\left(\delta ,\phi_{a}\right) & \overset{\mathrm{def}}{=} & \underset{d\leftarrow \delta }{E}\;\underset{a\leftarrow \phi_{a}\left(d\right)}{E}u_{a}\left(d,a\right) & = & \sum_{d\in D}\delta \left(d\right)\sum_{a\in A}\phi_{a}\left(d\right)\left(a\right)\;u_{a}\left(d,a\right) \end{array}\right.\\ \mathrm{Mixed}\;\mathrm{case}:\;\; & \left\{\begin{array}{ccccc} U_{d}\left(\delta ,\sigma_{a}\right) & \overset{\mathrm{def}}{=} & \underset{\begin{array}{c} d\leftarrow \delta \\ s_{a}\leftarrow \sigma_{a} \end{array}}{E}u_{d}\left(d,s_{a}\left(d\right)\right) & = & \sum_{\begin{array}{c} d\in D\\ s_{a}:D\;\to \;A \end{array}}\delta \left(d\right)\sigma_{a}\left(s_{a}\right)u_{d}\left(d,s_{a}\left(d\right)\right)\\ U_{a}\left(\delta ,\sigma_{a}\right) & \overset{\mathrm{def}}{=} & \underset{\begin{array}{c} d\leftarrow \delta \\ s_{a}\leftarrow \sigma_{a} \end{array}}{E}u_{a}\left(d,s_{a}\left(d\right)\right) & = & \sum_{\begin{array}{c} d\in D\\ s_{a}:D\;\to \;A \end{array}}\delta \left(d\right)\sigma_{a}\left(s_{a}\right)u_{a}\left(d,s_{a}\left(d\right)\right) \end{array}\right. \end{array}$$

The case of imperfect information is typically formalized by assuming an indistinguishability (equivalence) relation over the actions chosen by the leader, representing a scenario in which the follower cannot distinguish between the actions belonging to the same equivalence class. The pure strategies of the followers, therefore, are functions from the set of the equivalence classes on the actions of the leader to his/her own actions. Formally, a defender-first sequential game with imperfect information is a tuple $\left(D,\;K_{a}\to A,\;u_{d},u_{a}\right)$ where D, A, $u_{d}$ and $u_{a}$ are defined as in simultaneous games, and $K_{a}$ is a partition of D. The expected payoff functions are defined as before, except that now the argument of $\phi_{a}$ and $s_{a}$ is the equivalence class of *d*. Note that in the case in which all defender’s actions are indistinguishable from each other in the eyes of the attacker (totally imperfect information), we have $K_{a}=\left\{D\right\}$, and the expected payoff functions coincide with those of the simultaneous games. In contrast, in the games in which all defender’s actions are distinguishable from the viewpoint of the attacker (perfect information), we have $K_{a}=\left\{\left\{d\right\}|d\in D\right\}$.

In the standard game theory, under the assumption of perfect recall (i.e., the players never forget what they have learned), behavioral and mixed strategies are equivalent, in the sense that for any behavioral strategy, there is a mixed strategy that yields the same payoff, and vice versa. This is true for both cases of perfect and imperfect information; see [15], Chapter 11.4. In our leakage games, however, this equivalence does not hold anymore, as will be shown in Section 5 and Section 6.

#### 2.1.4. Zero-Sum Games and the Minimax Theorem

A game $\left(D,\;A,\;u_{d},u_{a}\right)$ is zero-sum if for any d∈D and any a∈A, the defender’s loss is equivalent to the attacker’s gain, i.e., $u_{d}\left(d,a\right)=-u_{a}\left(d,a\right)$. For brevity, in zero-sum games, we denote by *u* the attacker’s payoff function $u_{a}$ and by *U* the attacker’s expected payoff $U_{a}$ (Conventionally in game theory, the payoff *u* is set to be that of the first player, but we prefer to look at the payoff from the point of view of the attacker to be in line with the definition of payoff as vulnerability.). Consequently, the goal of the defender is to minimize *U*, and the goal of the attacker is to maximize it.

In simultaneous zero-sum games, the Nash equilibrium corresponds to the solution of the minimax problem (or equivalently, the maximin problem), namely the strategy profile $\left(\delta^{*},\alpha^{*}\right)$ such that $U\left(\delta^{*},\alpha^{*}\right)=\min_{\delta }\max_{\alpha }U\left(\delta ,\alpha \right)$. The von Neumann’s minimax theorem, in fact, ensures that such a solution (which always exists) is stable.

**Theorem** **1** (von Neumann’s minimax theorem)**.** *Let $X\subset R^{m}$ and $Y\subset R^{n}$ be compact convex sets, and U:X×Y→R be a continuous function such that U(x,y) is a convex function in x∈X and a concave function in y∈Y. Then:*

$$\min_{x\in X}\max_{y\in Y}U\left(x,y\right)=\max_{y\in Y}\min_{x\in X}U\left(x,y\right).$$

A related property is that, under the conditions of Theorem 1, there exists a saddle point $\left(x^{*},y^{*}\right)$ s.t., for all x∈X and y∈Y: $U\left(x^{*},y\right)\leq U\left(x^{*},y^{*}\right)\leq U\left(x,y^{*}\right)$.

The solution of the minimax problem can be obtained by using convex optimization techniques. In the case U(x,y) is affine in *x* and in *y*, we can also use linear optimization.

In the case D and A contain two elements each, there is a closed form for the solution. Let $D=\{d_{0},d_{1}\}$ and $A=\{a_{0},a_{1}\}$, respectively. Let $u_{ij}$ be the payoff of the defender on $d_{i},a_{j}$. Then, the Nash equilibrium $\left(\delta^{*},\alpha^{*}\right)$ is given by:

(1) $$\delta^{*}\left(d_{0}\right)=\frac{u_{11}-u_{10}}{u_{00}-u_{01}-u_{10}+u_{11}}\;\;\;\;\alpha^{*}\left(a_{0}\right)=\frac{u_{11}-u_{01}}{u_{00}-u_{01}-u_{10}+u_{11}}$$

if these values are in [0,1]. Note that, since there are only two elements, the strategy $\delta^{*}$ is completely specified by its value in $d_{0}$ and analogously for $\alpha^{*}$.

### 2.2. Quantitative Information Flow

Finally, we briefly review the standard framework of quantitative information flow, which is concerned with measuring the amount of information leakage in a (computational) system.

#### 2.2.1. Secrets and Vulnerability

A secret is some piece of sensitive information the defender wants to protect, such as a user’s password, social security number or current location. The attacker usually only has some partial knowledge about the value of a secret, represented as a probability distribution on secrets called a prior. We denote by X the set of possible secrets, and we typically use π to denote a prior belonging to the set DX of probability distributions over X.

The vulnerability of a secret is a measure of the payoff that it represents for the attacker. In this paper, we consider a very general notion of vulnerability, following [8], and we define a vulnerability V to be any continuous and convex function of type DX→R. It has been shown in [8] that these functions coincide with the set of *g*-vulnerabilities, and are, in a precise sense, the most general information measures w.r.t. a set of fundamental information-theoretic axioms (more precisely, if posterior vulnerability is defined as the expectation of the vulnerability of posterior distributions, the measure respects the fundamental information-theoretic properties of data-processing inequality (i.e., that post-processing can never increase information, but only destroy it) and of non-negativity of leakage (i.e., that by observing the output of a channel, an actor cannot, on average, lose information) if, and only if, vulnerability is convex). This notion, hence, subsumes all information measures that respect such fundamental principles, including the widely-adopted measures of Bayes vulnerability (also known as min-vulnerability, also known as (the converse of) Bayes risk) [9,10], Shannon entropy [11], guessing entropy [12] and *g*-vulnerability [13].

#### 2.2.2. Channels, Posterior Vulnerability and Leakage

Computational systems can be modeled as information theoretic channels. A channel C:X×Y→R is a function in which X is a set of input values, Y is a set of output values and C(x,y) represents the conditional probability of the channel producing output y∈Y when input x∈X is provided. Every channel *C* satisfies 0≤C(x,y)≤1 for all x∈X and y∈Y, and $\sum_{y\in Y}C\left(x,y\right)=1$ for all x∈X.

A distribution π∈DX and a channel *C* with inputs X and outputs Y induce a joint distribution p(x,y)=π(x)C(x,y) on X×Y, producing joint random variables X,Y with marginal probabilities $p\left(x\right)=\sum_{y}p\left(x,y\right)$ and $p\left(y\right)=\sum_{x}p\left(x,y\right)$, and conditional probabilities p(x∣y)=p(x,y)p(y) if p(y)≠0. For a given *y* (s.t. p(y)≠0), the conditional probabilities p(x∣y) for each x∈X form the posterior distribution $p_{X|y}$.

A channel *C* in which X is a set of secret values and Y is a set of observable values produced by a system can be used to model computations on secrets. Assuming the attacker has prior knowledge π about the secret value, knows how a channel *C* works and can observe the channel’s outputs, the effect of the channel is to update the attacker’s knowledge from π to a collection of posteriors $p_{X|y}$, each occurring with probability p(y).

Given a vulnerability V, a prior π and a channel *C*, the posterior vulnerability $V\left[\pi ,C\right]$ is the vulnerability of the secret after the attacker has observed the output of the channel *C*. Formally: $V\left[\pi ,C\right]\overset{\mathrm{def}}{=}\sum_{y\in Y}p\left(y\right)V\left[p_{X|y}\right]$.

Consider, for instance, the example of the password-checker with a timing side-channel from the Introduction (Example 4, also discussed in detail in Section 7). Here, the set of secrets X consists of all possible passwords (say, all strings of *n* bits), and a natural vulnerability function is Bayes-vulnerability, given by $V\left(\pi \right)=\max_{x\in X}\pi \left(x\right)$. This function expresses the adversary’s probability of guessing correctly the password in one try; assuming that the passwords are chosen uniformly, i.e., π is uniform, any guess would be correct with probability $2^{-n}$, giving $V\left(\pi \right)=2^{-n}$. Now, imagine that the timing side-channel reveals that the adversary’s input failed on the first bit. The adversary now knows the first bit of the password (say 0); hence, the posterior $p_{X|y}$ assigns probability zero to all passwords with first bit one and probability $2^{-(n-1)}$ to all passwords with first bit zero. This happens for all possible posteriors, giving posterior vulnerability $V\left[\pi ,C\right]=2^{-(n-1)}$ (two-times greater than the prior V).

It is known from the literature [8] that the posterior vulnerability is a convex function of π. Namely, for any channel *C*, any family of distributions $\left\{\pi_{i}\right\}$ and any set of convex coefficients $\left\{c_{i}\right\}$, we have:

$$\begin{array}{cc} V\left[\sum_{i}c_{i}\pi_{i},C\right]\leq & \;\sum_{i}c_{i}V\left[\pi_{i},C\right] \end{array}$$

The (information) leakage of channel *C* under prior π is a comparison between the vulnerability of the secret before the system was run (called prior vulnerability) and the posterior vulnerability of the secret. Leakage reflects how much the observation of the system’s outputs increases the attacker’s information about the secret. It can be defined either additively ($V\left[\pi ,C\right]-V\left[\pi \right]$) or multiplicatively (Vπ,CVπ). In the password-checker example, the additive leakage is $2^{-(n-1)}-2^{-n}=2^{-n}$, and the multiplicative leakage is 2−(n−1)2−n=2.

## 3. An Illustrative Example

We introduce an example that will serve as a running example throughout the paper. Although admittedly contrived, this example is simple and yet produces different leakage measures for all different combinations of visible/hidden choice and simultaneous/sequential games, thus providing a way to compare all different scenarios in which we are interested.

**Figure 1:**
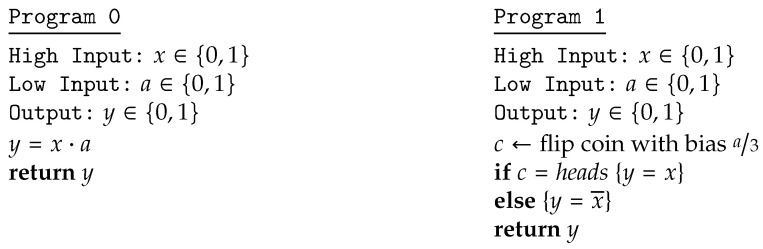
Alternative programs for the running example.

Consider that a binary secret must be processed by a program. As usual, a defender wants to protect the secret value, whereas an attacker wants to infer it by observing the system’s output. Assume the defender can choose which among two alternative versions of the program to run. Both programs take the secret value *x* as high input and a binary low input *a* whose value is chosen by the attacker. They both return the output in a low variable *y* (we adopt the usual convention in QIF of referring to secret variables, inputs and outputs in programs as high and to their observable counterparts as low). Program 0 returns the binary product of *x* and *a*, whereas Program 1 flips a coin with bias a3 (i.e., a coin that returns heads with probability a3) and returns *x* if the result is heads and the complement $\bar{x}$ of *x* otherwise. The two programs are represented in Figure 1.

The combined choices of the defender’s and of the attacker’s determine how the system behaves. Let D={0,1} represent the set of the defender’s choices, i.e., the index of the program to use, and A={0,1} represent the set of the attacker’s choices, i.e., the value of the low input *a*. We shall refer to the elements of D and A as actions. For each possible combination of actions d∈D and a∈A, we can construct a channel $C_{da}$ modeling how the resulting system behaves. Each channel $C_{da}$ is a function of type X×Y→R, where X={0,1} is the set of possible high input values for the system and Y={0,1} is the set of possible output values from the system. Intuitively, each channel provides the probability that the system (which was fixed by the defender) produces output y∈Y given that the high input is x∈X (and that the low input was fixed by the attacker). The four possible channels are depicted in Table 1.

Note that channel $C_{00}$ does not leak any information about the input *x* (i.e., it is non-interferent), whereas channels $C_{01}$ and $C_{10}$ completely reveal *x*. Channel $C_{11}$ is an intermediate case: it leaks some information about *x*, but not all.

We want to investigate how the defender’s and the attacker’s choices influence the leakage of the system. For that, we can just consider the (simpler) notion of posterior vulnerability, since in order to make the comparison fair, we need to assume that the prior is always the same in the various scenarios, and this implies that the leakage is in a one-to-one correspondence with the posterior vulnerability (this happens for both additive and multiplicative leakage).

For this example, assume we are interested in Bayes vulnerability [9,10], defined as $V\left(\pi \right)\;=\;\max_{x}\pi \left(x\right)$ for every π∈DX. Assume for simplicity that the prior is the uniform prior $\pi_{u}$. In this case, we know from [16] that the posterior Bayes vulnerability of a channel is the sum of the greatest elements of each column, divided by the total number of inputs. Table 2 provides the Bayes vulnerability $V_{da}\overset{\mathrm{def}}{=}V\left[\pi_{u},C_{da}\right]$ of each channel considered above.

Naturally, the attacker aims at maximizing the vulnerability of the system, while the defender tries to minimize it. The resulting vulnerability will depend on various factors, in particular on whether the two players make their choice simultaneously (i.e., without knowing the choice of the opponent) or sequentially. Clearly, if the choice of a player who moves first is known by an opponent who moves second, the opponent will be at an advantage. In the above example, for instance, if the defender knows the choice *a* of the attacker, the most convenient choice for him/her is to set d=a, and the vulnerability will be at most 23. The other way around, if the attacker knows the choice *d* of the defender, the most convenient choice for him/her is to set a≠d. The vulnerability in this case will be one.

Things become more complicated when players make choices simultaneously. None of the pure choices of *d* and *a* are the best for the corresponding player, because the vulnerability of the system depends also on the (unknown) choice of the other player. Yet, there is a strategy leading to the best possible situation for both players (the Nash equilibrium), but it is mixed (i.e., probabilistic), in that the players randomize their choices according to some precise distribution.

Another factor that affects vulnerability is whether or not the defender’s choice is known to the attacker at the moment in which he/she observes the output of the channel. Obviously, this corresponds to whether or not the attacker knows what channel he/she is observing. Both cases are plausible: naturally, the defender has all the interest in keeping his/her choice (and hence, the channel used) secret, since then, the attack will be less effective (i.e., leakage will be smaller). On the other hand, the attacker may be able to identify the channel used anyway, for instance because the two programs have different running times. We will call these two cases hidden and visible choice, respectively.

It is possible to model players’ strategies, as well as hidden and visible choices, as operations on channels. This means that we can look at the whole system as if it were a single channel, which will turn out to be useful for some proofs of our technical results. The next section is dedicated to the definition of these operators. We will calculate the exact values for our example in Section 5.

## 4. Choice Operators for Protocol Composition

In this section, we define the operators of visible and hidden choice for protocol composition. These operators are formally defined on the channel matrices of the protocols, and since channels are a particular kind of matrix, we use these matrix operations to define the operations of visible and hidden choice among channels and to prove important properties of these channel operations.

### 4.1. Matrices and Their Basic Operators

Given two sets X and Y, a matrix is a total function of type X×Y→R. Two matrices $M_{1}:\;X_{1}\;\times \;Y_{1}\to R$ and $M_{2}:X_{2}\times Y_{2}\to R$ are said to be compatible if $X_{1}=X_{2}$. If it is also the case that $Y_{1}=Y_{2}$, we say that the matrices have the same type. The scalar multiplication r·M between a scalar *r* and a matrix *M* is defined as usual, and so is the summation $\left(\sum_{i\in I}M_{i}\right)\left(x,y\right)=M_{i_{1}}\left(x,y\right)+\ldots +M_{i_{n}}\left(x,y\right)$ of a family ${\left\{M_{i}\right\}}_{i\in I}$ of matrices all of a same type.

Given a family ${\left\{M_{i}\right\}}_{i\in I}$ of compatible matrices s.t. each $M_{i}$ has type $X\times Y_{i}\to R$, their concatenation ${⋄}_{i\in I}$ is the matrix having all columns of every matrix in the family, in such a way that every column is tagged with the matrix from which it came. Formally, $\left({⋄}_{i\in I}M_{i}\right)\left(x,\left(y,j\right)\right)=\;M_{j}\left(x,y\right)$, if $y\in Y_{j}$, and the resulting matrix has type $X\times \left({⨆}_{i\in I}Y_{i}\right)\to R$. (We use ${⨆}_{i\in I}Y_{i}=Y_{i_{1}}⊔Y_{i_{2}}⊔\ldots ⊔Y_{i_{n}}$ to denote the disjoint union $\left\{\left(y,i\right)|y\in Y_{i},i\in I\right\}$ of the sets $Y_{i_{1}}$, $Y_{i_{2}}$, …, $Y_{i_{n}}$.) When the family $\left\{M_{i}\right\}$ has only two elements we may use the binary version ◊ of the concatenation operator. The following depicts the concatenation of two matrices $M_{1}$ and $M_{2}$ in tabular form.

M1y1y2x112x234⋄M2y1y2y3x1567x28910=M1⋄M2(y1,1)(y2,1)(y1,2)(y2,2)(y3,2)x112567x2348910

### 4.2. Channels and Their Hidden and Visible Choice Operators

A channel is a stochastic matrix, i.e., all elements are non-negative, and all rows sum up to one. Here, we will define two operators specific for channels. In the following, for any real value 0≤p≤1, we denote by $\bar{p}$ the value 1−p.

#### 4.2.1. Hidden Choice

The first operator models a hidden probabilistic choice among channels. Consider a family ${\left\{C_{i}\right\}}_{i\in I}$ of channels of the same type. Let μ∈DI be a probability distribution on the elements of the index set I. Consider an input *x* is fed to one of the channels in ${\left\{C_{i}\right\}}_{i\in I}$, where the channel is randomly picked according to μ. More precisely, an index i∈I is sampled with probability μ(i), then the input *x* is fed to channel $C_{i}$, and the output *y* produced by the channel is then made visible, but not the index *i* of the channel that was used. Note that we consider hidden choice only among channels of the same type: if the sets of outputs were not identical, the produced output might implicitly reveal which channel was used.

Formally, given a family ${\left\{C_{i}\right\}}_{i\in I}$ of channels s.t. each $C_{i}$ has same type X×Y→R, the hidden choice operator ${⨊}_{i\leftarrow \mu }$ is defined as ${⨊}_{i\leftarrow \mu }C_{i}=\sum_{i\in I}\mu \left(i\right)\;C_{i}$.

**Proposition** **1** (Type of hidden choice)**.** Given a family ${\left\{C_{i}\right\}}_{i\in I}$ of channels of type X×Y→R, and a distribution μ on I, the hidden choice ${⨊}_{i\leftarrow \mu }C_{i}$ is a channel of type X×Y→R.

See Appendix A for the proof.

In the particular case in which the family $\left\{C_{i}\right\}$ has only two elements $C_{i_{1}}$ and $C_{i_{2}}$, the distribution μ on indexes is completely determined by a real value 0≤p≤1 s.t. $\mu (i_{1})=p$ and $\mu \left(i_{2}\right)=\bar{p}$. In this case, we may use the binary version $\;{}_{p}⊕\;$ of the hidden choice operator: $C_{i_{1}}\;{}_{p}⊕\;C_{i_{2}}=p\;C_{i_{1}}+\bar{p}\;C_{i_{2}}$. The example below depicts the hidden choice between channels $C_{1}$ and $C_{2}$, with probability p=13.

C1y1y2x11212x2132313⊕C2y1y2x11323x21212=C113⊕C2y1y2x17181118x24959

#### 4.2.2. Visible Choice

The second operator models a visible probabilistic choice among channels. Consider a family ${\left\{C_{i}\right\}}_{i\in I}$ of compatible channels. Let μ∈DI be a probability distribution on the elements of the index set I. Consider an input *x* is fed to one of the channels in ${\left\{C_{i}\right\}}_{i\in I}$, where the channel is randomly picked according to μ. More precisely, an index i∈I is sampled with probability μ(i), then the input *x* is fed to channel $C_{i}$, and the output *y* produced by the channel is then made visible, along with the index *i* of the channel that was used. Note that visible choice makes sense only between compatible channels, but it is not required that the output set of each channel be the same.

Formally, given ${\left\{C_{i}\right\}}_{i\in I}$ of compatible channels s.t. each $C_{i}$ has type $X\times Y_{i}\to R$, and a distribution μ on I, the visible choice operator ${\left\lfloor \;\cdot \;\right\rfloor }_{i\leftarrow \mu }$ is defined as ${\left\lfloor \;\cdot \;\right\rfloor }_{i\leftarrow \mu }C_{i}={⋄}_{i\in I}\;\mu \left(i\right)\;C_{i}$.

**Proposition** **2** (Type of visible choice)**.** *Given a family ${\left\{C_{i}\right\}}_{i\in I}$ of compatible channels s.t. each $C_{i}$ has type $X\times Y_{i}\to R$ and a distribution μ on I, the result of the visible choice ${\left\lfloor \;\cdot \;\right\rfloor }_{i\leftarrow \mu }C_{i}$ is a channel of type $X\times \left({⨆}_{i\in I}Y_{i}\right)\to R$.*

See Appendix A for the proof.

In the particular case that the family $\left\{C_{i}\right\}$ has only two elements $C_{i_{1}}$ and $C_{i_{2}}$, the distribution μ on indexes is completely determined by a real value 0≤p≤1 s.t. $\mu (i_{1})=p$ and $\mu \left(i_{2}\right)=\bar{p}$. In this case, we may use the binary version $\;{}_{p}\left\lfloor \;\cdot \;\right\rfloor \;$ of the visible choice operator: $C_{i_{1}}\;{}_{p}\left\lfloor \;\cdot \;\right\rfloor \;C_{i_{2}}=p\;C_{i_{1}}⋄\bar{p}\;C_{i_{2}}$. The following depicts the visible choice between channels $C_{1}$ and $C_{3}$, with probability p=13.

C1y1y2x11212x2132313⌊·⌋C3y1y3x11323x21212=C113⌊·⌋C3(y1,1)(y2,1)(y1,3)(y3,3)x116162949x219291313

### 4.3. Properties of Hidden and Visible Choice Operators

We now prove algebraic properties of channel operators. These properties will be useful when we model a (more complex) protocol as the composition of smaller channels via hidden or visible choice.

Whereas the properties of hidden choice hold generally with equality, those of visible choice are subtler. For instance, visible choice is not idempotent, since in general $C\;{}_{p}\left\lfloor \;\cdot \;\right\rfloor \;C\neq C$ (in fact, if *C* has type X×Y→R, $C\;{}_{p}\left\lfloor \;\cdot \;\right\rfloor \;C$ has type X×(Y⊔Y)→R). However, idempotency and other properties involving visible choice hold if we replace the notion of equality with the more relaxed notion of “equivalence” between channels. Intuitively, two channels are equivalent if they have the same input space and yield the same value of vulnerability for every prior and every vulnerability function.

**Definition** **1** (Equivalence of channels)**.** Two compatible channels $C_{1}$ and $C_{2}$ with domain X are equivalent, denoted by $C_{1}\approx C_{2}$, if for every prior π∈DX and every posterior vulnerability V, we have $V\left[\pi ,C_{1}\right]\;=\;V\left[\pi ,C_{2}\right]$.

Two equivalent channels are indistinguishable from the point of view of information leakage, and in most cases, we can just identify them. Indeed, nowadays, there is a tendency to use abstract channels [8,17], which capture exactly the important behavior with respect to any form of leakage. In this paper, however, we cannot use abstract channels because the hidden choice operator needs a concrete representation in order to be defined unambiguously.

The first properties we prove regard idempotency of operators, which can be used do simplify the representation of some protocols.

**Proposition** **3** (Idempotency)**.** *Given a family ${\left\{C_{i}\right\}}_{i\in I}$ of channels s.t. $C_{i}=C$ for all i∈I, and a distribution μ on I, then: (a) ${⨊}_{i\leftarrow \mu }C_{i}=C$; and (b) ${\left\lfloor \;\cdot \;\right\rfloor }_{i\leftarrow \mu }C_{i}\approx C$.*

See Appendix A for the proof.

The following properties regard the reorganization of operators, and they will be essential in some technical results in which we invert the order in which hidden and visible choice are applied in a protocol.

**Proposition** **4** (“Reorganization of operators”)**.** *Given a family ${\left\{C_{ij}\right\}}_{i\in I,j\in J}$ of channels indexed by sets I and J, a distribution μ on I and a distribution η on J*:

*(a)*  ${⨊}_{i\leftarrow \mu }\;{⨊}_{j\leftarrow \eta }C_{ij}={⨊}_{\begin{array}{c} i\leftarrow \mu \\ j\leftarrow \eta \end{array}}C_{ij}$, if all $C_{i}$’s have the same type;
*(b)*  ${\left\lfloor \;\cdot \;\right\rfloor }_{i\leftarrow \mu }\;{\left\lfloor \;\cdot \;\right\rfloor }_{j\leftarrow \eta }C_{ij}\approx {\left\lfloor \;\cdot \;\right\rfloor }_{\begin{array}{c} i\leftarrow \mu \\ j\leftarrow \eta \end{array}}C_{ij}$, if all $C_{i}$’s are compatible; and
*(c)*  ${⨊}_{i\leftarrow \mu }\;{\left\lfloor \;\cdot \;\right\rfloor }_{j\leftarrow \eta }C_{ij}\approx {\left\lfloor \;\cdot \;\right\rfloor }_{j\leftarrow \eta }\;{⨊}_{i\leftarrow \mu }C_{ij}$, if, for each *i*, all $C_{ij}$’s have the same type $X\times Y_{j}\to R$.

See Appendix A for the proof.

Finally, analogous properties of the binary operators are shown in Appendix B.

### 4.4. Properties of Vulnerability w.r.t. Channel Operators

We now derive some relevant properties of vulnerability w.r.t. our channel operators, which will be later used to obtain the Nash equilibria in information leakage games with different choice operations.

The first result states that posterior vulnerability is convex w.r.t. hidden choice (this result was already presented in [6]) and linear w.r.t. to visible choice.

**Theorem** **2** (Convexity/linearity of posterior vulnerability w.r.t. choices)**.** *Let ${\left\{C_{i}\right\}}_{i\in I}$ be a family of channels and μ be a distribution on I. Then, for every distribution π on X and every vulnerability* V:

*1*. posterior vulnerability is convex w.r.t. to hidden choice: $V\left[\pi ,{⨊}_{i\leftarrow \mu }C_{i}\right]\leq \sum_{i\in I}\mu \left(i\right)\;V\left[\pi ,C_{i}\right]$ if all $C_{i}$’s have the same type.
*2*. posterior vulnerability is linear w.r.t. to visible choice: $V\left[\pi ,{\left\lfloor \;\cdot \;\right\rfloor }_{i\leftarrow \mu }C_{i}\right]=\sum_{i\in I}\mu \left(i\right)\;V\left[\pi ,C_{i}\right]$ if all $C_{i}$’s are compatible.

**Proof.** 

1. Let us call X×Y→R the type of each channel $C_{i}$ in the family $\left\{C_{i}\right\}$. Then:
  $$\begin{array}{ccc} V\left[\pi ,\underset{i\leftarrow \mu }{⨊}C_{i}\right]= & \;V\left[\pi ,\sum_{i}\mu \left(i\right)C_{i}\right] & \left(\mathrm{by}\;\mathrm{the}\;\mathrm{definition}\;\mathrm{of}\;\mathrm{hidden}\;\mathrm{choice}\right)\\ = & \;\sum_{y\in Y}p\left(y\right)\cdot V\left[\frac{\pi \left(\cdot \right)\sum_{i}\mu \left(i\right)C_{i}\left(\cdot ,y\right)}{p(y)}\right] & \left(\mathrm{by}\;\mathrm{the}\;\mathrm{definition}\;\mathrm{of}\;\mathrm{posterior}\;V\right)\\ = & \;\sum_{y\in Y}p\left(y\right)\cdot V\left[\sum_{i}\mu \left(i\right)\frac{\pi \left(\cdot \right)C_{i}\left(\cdot ,y\right)}{p(y)}\right] & \\ \leq & \;\sum_{y\in Y}p\left(y\right)\cdot \sum_{i}\mu \left(i\right)V\left[\frac{\pi \left(\cdot \right)C_{i}\left(\cdot ,y\right)}{p(y)}\right] & \left(\mathrm{by}\;\mathrm{the}\;\mathrm{convexity}\;\mathrm{of}\;V\right)\\ = & \;\sum_{i}\mu \left(i\right)\sum_{y\in Y}p\left(y\right)V\left[\frac{\pi \left(\cdot \right)C_{i}\left(\cdot ,y\right)}{p(y)}\right] & \\ = & \;\sum_{i}\mu \left(i\right)V\left[\pi ,C_{i}\right] & \end{array}$$
  where $p\left(y\right)=\sum_{x\in X}\pi \left(x\right)\sum_{i}\mu \left(i\right)C_{i}\left(x,y\right)$.
2. Let us call $X\times Y_{i}\to R$ the type of each channel $C_{i}$ in the family $\left\{C_{i}\right\}$. Then:
  $$\begin{array}{ccc} V\left[\pi ,{\left\lfloor \;\cdot \;\right\rfloor }_{i\leftarrow \mu }C_{i}\right]= & \;V\left[\pi ,{⋄}_{i}\mu \left(i\right)C_{i}\right] & \left(\mathrm{by}\;\mathrm{the}\;\mathrm{definition}\;\mathrm{of}\;\mathrm{visible}\;\mathrm{choice}\right)\\ = & \;\sum_{y\in Y}p\left(y\right)\cdot V\left[\frac{\pi (\cdot ){⋄}_{i}\mu \left(i\right)C_{i}\left(\cdot ,y\right)}{p(y)}\right] & \left(\mathrm{by}\;\mathrm{the}\;\mathrm{definition}\;\mathrm{of}\;\mathrm{posterior}\;V\right)\\ = & \;\sum_{y\in Y}p\left(y\right)\cdot V\left[{⋄}_{i}\mu \left(i\right)\frac{\pi \left(\cdot \right)C_{i}\left(\cdot ,y\right)}{p(y)}\right] & \\ = & \;\sum_{y\in Y}p\left(y\right)\cdot \sum_{i}\mu \left(i\right)V\left[\frac{\pi \left(\cdot \right)C_{i}\left(\cdot ,y\right)}{p(y)}\right] & \left(\mathrm{see}\;(*)\;\mathrm{below}\right)\\ = & \;\sum_{i}\mu \left(i\right)\sum_{y\in Y}p\left(y\right)V\left[\frac{\pi \left(\cdot \right)C_{i}\left(\cdot ,y\right)}{p(y)}\right] & \\ = & \;\sum_{i}\mu \left(i\right)V\left[\pi ,C_{i}\right] & \end{array}$$
  where $p\left(y\right)=\sum_{x\in X}\pi \left(x\right)\sum_{i}\mu \left(i\right)C_{i}\left(x,y\right)$, and step (*) holds because in the vulnerability of a concatenation of matrices, every column will contribute to the vulnerability in proportion to its weight in the concatenation; hence, it is possible to break the vulnerability of a concatenated matrix as the weighted sum of the vulnerabilities of its sub-matrices.

 ☐

The next result is concerned with posterior vulnerability under the composition of channels using both operators.

**Corollary** **1** (Convex-linear payoff function)**.** Let ${\left\{C_{ij}\right\}}_{i\in I,j\in J}$ be a family of channels, all with domain X and with the same type, and let π∈DX, and V be any vulnerability. Define U:DI×DJ→R as follows: $U\left(\mu ,\eta \right)\overset{\mathrm{def}}{=}V\left[\pi ,{⨊}_{i\leftarrow \mu }\;{\left\lfloor \;\cdot \;\right\rfloor }_{j\leftarrow \eta }\;C_{ij}\right]$. Then, U is convex on μ and linear on η.

**Proof.**  To see that U(μ,η) is convex on μ, note that:

$$\begin{array}{ccc} U(\mu ,\eta )= & \;V\left[\pi ,\underset{i\leftarrow \mu }{⨊}\;\underset{j\leftarrow \eta }{\left\lfloor \;\cdot \;\right\rfloor }\;C_{ij}\right] & \left(\mathrm{by}\;\mathrm{definition}\right)\\ \leq & \;\sum_{i}\mu \left(i\right)\;V\left[\pi ,\underset{j\leftarrow \eta }{\left\lfloor \;\cdot \;\right\rfloor }\;C_{ij}\right] & \left(\mathrm{by}\;\mathrm{Theorem}\;2\right) \end{array}$$

To see that U(μ,η) is linear on η, note that:

$$\begin{array}{ccc} U(\mu ,\eta )= & \;V\left[\pi ,\underset{i\leftarrow \mu }{⨊}\;\underset{j\leftarrow \eta }{\left\lfloor \;\cdot \;\right\rfloor }\;C_{ij}\right] & \left(\mathrm{by}\;\mathrm{definition}\right)\\ = & \;V\left[\pi ,\underset{j\leftarrow \eta }{\left\lfloor \;\cdot \;\right\rfloor }\;\underset{i\leftarrow \mu }{⨊}\;C_{ij}\right] & \left(\mathrm{by}\;\mathrm{Proposition}\;4\right)\\ = & \;\sum_{j}\eta \left(j\right)\;V\left[\pi ,\underset{i\leftarrow \mu }{⨊}\;C_{ij}\right] & \left(\mathrm{by}\;\mathrm{Theorem}\;2\right) \end{array}$$

 ☐

## 5. Information Leakage Games

In this section, we present our framework for reasoning about information leakage, extending the notion of information leakage games proposed in [6] from only simultaneous games with hidden choice to both simultaneous and sequential games, with either hidden or visible choice.

In an information leakage game, the defender tries to minimize the leakage of information from the system, while the attacker tries to maximize it. In this basic scenario, their goals are just opposite (zero-sum). Both of them can influence the execution and the observable behavior of the system via a specific set of actions. We assume players to be rational (i.e., they are able to figure out what is the best strategy to maximize their expected payoff) and that the set of actions and the payoff function are common knowledge.

Players choose their own strategy, which in general may be probabilistic (i.e., behavioral or mixed) and choose their action by a random draw according to that strategy. After both players have performed their actions, the system runs and produces some output value, which is visible to the attacker and may leak some information about the secret. The amount of leakage constitutes the attacker’s gain and the defender’s loss.

To quantify the leakage, we model the system as an information-theoretic channel (cf. Section 2.2). We recall that leakage is defined as the difference (additive leakage) or the ratio (multiplicative leakage) between posterior and prior vulnerability. Since we are only interested in comparing the leakage of different channels for a given prior, we will define the payoff just as the posterior vulnerability, as the value of prior vulnerability will be the same for every channel.

### 5.1. Defining Information Leakage Games

A (information) leakage game consists of:

(1) two nonempty sets D, A of defender’s and attacker’s actions, respectively,
(2) a function C:D×A→(X×Y→R) that associates with each pair of actions (d,a)∈D×A a channel $C_{da}:X\times Y\to R$,
(3) a prior π∈DX on secrets and
(4) a vulnerability measure V, used to define the payoff function u:D×A→R for pure strategies as $u\left(d,a\right)\overset{\mathrm{def}}{=}V\left[\pi ,C_{da}\right]$. We have only one payoff function because the game is zero-sum.

Like in traditional game theory, the order of actions and the extent by which a player knows the move performed by the opponent play a critical role in deciding strategies and determining the payoff. In security, however, knowledge of the opponent’s move affects the game in yet another way: the effectiveness of the attack, i.e., the amount of leakage, depends crucially on whether or not the attacker knows what channel is being used. It is therefore convenient to distinguish two phases in the leakage game:

- Phase 1: determination of players’ strategies and the subsequent choice of their actions.
  Each player determines the most convenient strategy (which in general is probabilistic) for himself/herself, and draws his/her action accordingly. One of the players may commit first to his/her action, and his/her choice may or may not be revealed to the follower. In general, knowledge of the leader’s action may help the follower choose a more advantageous strategy.
- Phase 2: observation of the resulting channel’s output and payoff computation.
  The attacker observes the output of the selected channel $C_{da}$ and performs his/her attack on the secret. In case he/she knows the defender’s action, he/she is able to determine the exact channel $C_{da}$ being used (since, of course, the attacker knows his/her own action), and his/her payoff will be the posterior vulnerability $V\left[\pi ,C_{da}\right]$. However, if the attacker does not know exactly which channel has been used, then his/her payoff will be smaller.

Note that the issues raised in Phase 2 are typical of leakage games; they do not have a correspondence (to the best of our knowledge) in traditional game theory. Indeed, in traditional game theory, the resulting payoff is a deterministic function of all players’ actions. On the other hand, the extra level of randomization provided by the channel is central to security, as it reflects the principle of preventing the attacker from inferring the secret by obfuscating the link between the secret and observables.

Following the above discussion, we consider various possible scenarios for games, along two lines of classification. The first classification concerns Phase 1 of the game, in which strategies are selected and actions are drawn, and consists of three possible orders for the two players’ actions.

- Simultaneous.
  The players choose (draw) their actions in parallel, each without knowing the choice of the other.
- Sequential, defender-first.
  The defender draws an action, and commits to it, before the attacker does.
- Sequential, attacker-first.
  The attacker draws an action, and commits to it, before the defender does.

Note that these sequential games may present imperfect information (i.e., the follower may not know the leader’s action) and that we have to further specify whether we use behavioral or mixed strategies.

The second classification concerns Phase 2 of the game, in which some leakage occurs as a consequence of the attacker’s observation of the channel’s output and consists of two kinds of knowledge the attacker may have at this point about the channel that was used.

- Visible choice.
  The attacker knows the defender’s action when he/she observes the output of the channel, and therefore, he/she knows which channel is being used. Visible choice is modeled by the operator ⌊·⌋.
- Hidden choice.
  The attacker does not know the defender’s action when he/she observes the output of the channel, and therefore, in general, he/she does not exactly know which channel is used (although in some special cases, he/she may infer it from the output). Hidden choice is modeled by the operator ⨊.

Note that the distinction between sequential and simultaneous games is orthogonal to that between visible and hidden choice. Sequential and simultaneous games model whether or not, respectively, the follower’s choice can be affected by knowledge of the leader’s action. This dichotomy captures how knowledge about the other player’s actions can help a player choose his/her own action, and it concerns how Phase 1 of the game occurs. On the other hand, visible and hidden choice capture whether or not, respectively, the attacker is able to fully determine the channel representing the system, once the defender and attacker’s actions have already been fixed. This dichotomy reflects the different amounts of information leaked by the system as viewed by the attacker, and it concerns how Phase 2 of the game occurs. For instance, in a simultaneous game, neither player can choose his/her action based on the choice of the other. However, depending on whether or not the defender’s choice is visible, the attacker will or will not, respectively, be able to completely recover the channel used, which will affect the amount of leakage.

If we consider also the subdivision of sequential games into perfect and imperfect information, there are 10 possible different combinations. Some, however, make little sense. For instance, the defender-first sequential game with perfect information (by the attacker) does not combine naturally with hidden choice ⨊, since that would mean that the attacker knows the action of the defender and chooses his/her strategy accordingly, but forgets it at the moment of computing the channel and its vulnerability (we assume perfect recall, i.e., the players never forget what they have learned). Yet, other combinations are not interesting, such as the attacker-first sequential game with (totally) imperfect information (by the defender), since it coincides with the simultaneous-game case. Note that the attacker and defender are not symmetric with respect to hiding/revealing their actions *a* and *d*, since the knowledge of *a* affects the game only in the usual sense of game theory (in Phase 1), while the knowledge of *d* also affects the computation of the payoff (in Phase 2). Note that the attacker and defender are not symmetric with respect to hiding/revealing their actions *a* and *d*, since the knowledge of *a* affects the game only in the usual sense of game theory, while the knowledge of *d* also affects the computation of the payoff (cf. “Phase 2” above). Other possible combinations would come from the distinction between behavioral and mixed strategies, but, as we will see, they are always equivalent except in one scenario, so for the sake of conciseness, we prefer to treat it as a case apart.

Table 3 lists the meaningful and interesting combinations. In Game V, we assume imperfect information: the attacker does not know the action chosen by the defender. In all the other sequential games, we assume that the follower has perfect information. In the remainder of this section, we discuss each game individually, using the example of Section 3 as a running example.

#### 5.1.1. Game I (Simultaneous with Visible Choice)

This simultaneous game can be represented by a tuple (D,A,u). As in all games with visible choice ⌊·⌋, the expected payoff U of a mixed strategy profile (δ,α) is defined to be the expected value of *u*, as in traditional game theory:

$$\begin{array}{c} U\left(\delta ,\alpha \right)\overset{\mathrm{def}}{=}\underset{\begin{array}{c} d\leftarrow \delta \\ a\leftarrow \alpha \end{array}}{E}u\left(d,a\right)=\sum_{\begin{array}{c} d\in D\\ a\in A \end{array}}\delta \left(d\right)\;\alpha \left(a\right)\;u\left(d,a\right), \end{array}$$

where we recall that $u\left(d,a\right)=V\left[\pi ,C_{da}\right]$.

From Theorem 2 (2), we derive that $U\left(\delta ,\alpha \right)=V\left[\pi ,{\left\lfloor \;\cdot \;\right\rfloor }_{\begin{array}{c} d\leftarrow \delta \\ a\leftarrow \alpha \end{array}}C_{da}\right]$, and hence, the whole system can be equivalently regarded as the channel ${\left\lfloor \;\cdot \;\right\rfloor }_{\begin{array}{c} d\leftarrow \delta \\ a\leftarrow \alpha \end{array}}C_{da}$. Still from Theorem 2 (2), we can derive that U(δ,α) is linear in δ and α. Therefore the Nash equilibrium can be computed using the standard method (cf. Section 2.1).

**Example** **5.** Consider the example of Section 3 in the setting of Game I, with a uniform prior. The Nash equilibrium $\left(\delta^{*},\alpha^{*}\right)$ can be obtained using the closed formula from Section 2.1, and it is given by δ*(0)=α*(0)=(23−1)(12−1−1+23)=25. The corresponding payoff is U(δ*,α*)=252512+2535+3525+353523=45.

#### 5.1.2. Game II (Defender-First with Visible Choice)

This defender-first sequential game can be represented by a tuple (D,D→A,u). We will first consider mixed strategies for the follower (which in this case is the attacker), namely strategies of type D(D→A). Hence, a (mixed) strategy profile is of the form $\left(\delta ,\sigma_{a}\right)$, with δ∈DD and $\sigma_{a}\in D\left(D\;\to \;A\right)$, and the corresponding payoff is:

$$\begin{array}{c} U\left(\delta ,\sigma_{a}\right)\overset{\mathrm{def}}{=}\underset{\begin{array}{c} d\leftarrow \delta \\ s_{a}\leftarrow \sigma_{a} \end{array}}{E}u\left(d,s_{a}\left(d\right)\right)=\sum_{\begin{array}{c} d\in D\\ s_{a}:D\;\to \;A \end{array}}\delta \left(d\right)\;\sigma_{a}\left(s_{a}\right)\;u\left(d,s_{a}\left(d\right)\right), \end{array}$$

where $u\left(d,s_{a}\left(d\right)\right)=V\left[\pi ,C_{ds_{a}\left(d\right)}\right]$.

Again, from Theorem 2 (2), we derive: $U\left(\delta ,\sigma_{a}\right)=V\left[\pi ,{\left\lfloor \;\cdot \;\right\rfloor }_{\begin{array}{c} d\leftarrow \delta \\ s_{a}\leftarrow \sigma_{a} \end{array}}C_{ds_{a}\left(d\right)}\right]$, and hence, the system can be expressed as a channel ${\left\lfloor \;\cdot \;\right\rfloor }_{\begin{array}{c} d\leftarrow \delta \\ s_{a}\leftarrow \sigma_{a} \end{array}}C_{ds_{a}\left(d\right)}$. From the same theorem, we also derive that $U(\delta ,\sigma_{a})$ is linear in δ and $\sigma_{a}$, so the mutually optimal strategies can be obtained again by solving the minimax problem. In this case, however, the solution is particularly simple, because there are always deterministic optimal strategy profiles. We first consider the case of attacker’s strategies of type D(D→A).

**Theorem 3** (Pure-strategy Nash equilibrium in Game II: strategies of type D(D→A))**.** Consider a defender-first sequential game with visible choice and attacker’s strategies of type D(D→A). Let $d^{*}\;\overset{\mathrm{def}}{=}\;{\mathrm{argmin}}_{d}\max_{a}u\left(d,a\right)$, and let $s_{a}^{*}:D\;\to \;A$ be defined as $s_{a}^{*}\left(d\right)\overset{\mathrm{def}}{=}{\mathrm{argmax}}_{a}u\left(d,a\right)$ (if there is more than one a that maximizes u(d,a), we select one of them arbitrarily). Then, for every δ∈DD and $\sigma_{a}\in D\left(D\;\to \;A\right)$, we have $U\left(d^{*},\sigma_{a}\right)\leq u\left(d^{*},s_{a}^{*}\left(d^{*}\right)\right)\leq U\left(\delta ,s_{a}^{*}\right)$.

**Proof.**  Let δ and $\sigma_{a}$ be arbitrary elements of DD and D(D→A), respectively. Then:

$$\begin{array}{ccc} U(d^{*},\sigma_{a})= & \;\;\sum_{s_{a}:D\;\to \;A}\sigma_{a}\left(s_{a}\right)\;u\left(d^{*},s_{a}\left(d^{*}\right)\right) & \\ \leq & \;\;\sum_{s_{a}:D\;\to \;A}\sigma_{a}\left(s_{a}\right)\;u\left(d^{*},s_{a}^{*}\left(d^{*}\right)\right) & \left(\mathrm{by}\;\mathrm{the}\;\mathrm{definition}\;\mathrm{of}s_{a}^{*}\right)\\ = & \;\;u(d^{*},s_{a}^{*}\left(d^{*}\right)) & \left(\mathrm{since}\;\sigma_{a}\;\mathrm{is}\;a\;\mathrm{distribution}\right)\\ = & \;\;\sum_{d\in D}\delta \left(d\right)\;u\left(d^{*},s_{a}^{*}\left(d^{*}\right)\right) & \left(\mathrm{since}\;\delta \;\mathrm{is}\;a\;\mathrm{distribution}\right)\\ \leq & \;\;\sum_{d\in D}\delta \left(d\right)\;u\left(d,s_{a}^{*}\left(d\right)\right) & \left(\mathrm{by}\;\mathrm{the}\;\mathrm{definition}\;\mathrm{of}\;d^{*}\right)\\ = & \;\;U(\delta ,s_{a}^{*}) & \end{array}$$

 ☐

Hence, to find the optimal strategy, it is sufficient for the defender to find the action $d^{*}$ that minimizes $\max_{a}u\left(d^{*},a\right)$, while the attacker’s optimal choice is the pure strategy $s_{a}^{*}$ such that $s_{a}^{*}\left(d\right)\;=\;{\mathrm{argmax}}_{a}u\left(d,a\right)$, where *d* is the (visible) move by the defender.

**Example** **6.** Consider the example of Section 3 in the setting of Game II, with uniform prior. If the defender chooses zero, then the attacker chooses one. If the defender chooses one, then the attacker chooses zero. In both cases, the payoff is one. The game has therefore two solutions, $\left(\delta_{1}^{*},\alpha_{1}^{*}\right)$ and $\left(\delta_{2}^{*},\alpha_{2}^{*}\right)$, with $\delta_{1}^{*}\left(0\right)=1$, $\alpha_{1}^{*}\left(0\right)=0$ and $\delta_{2}^{*}\left(0\right)=0$, $\alpha_{2}^{*}\left(1\right)=1$.

Consider now the case of behavioral strategies. Following the same line of reasoning as before, we can see that under the strategy profile $\left(\delta ,\phi_{a}\right)$, the system can be expressed as the channel:

$$\underset{d\leftarrow \delta }{\left\lfloor \;\cdot \;\right\rfloor }\;\underset{a\leftarrow \phi_{a}\left(d\right)}{\left\lfloor \;\cdot \;\right\rfloor }C_{da}.$$

This is also in this case that there are deterministic optimal strategy profiles. An optimal strategy for the follower (in this case, the attacker) consists of looking at the action *d* chosen by the leader and then selecting with probability one the action *a* that maximizes u(d,a).

**Theorem 4** (Pure-strategy Nash equilibrium in Game II: strategies of type D→D(A))**.** Consider a defender-first sequential game with visible choice and attacker’s strategies of type D→D(A). Let $d^{*}\overset{\mathrm{def}}{=}{\mathrm{argmin}}_{d}\max_{a}u\left(d,a\right)$, and let $\phi_{a}^{*}:D\to D\left(A\right)$ be defined as $\phi_{a}^{*}\left(d\right)\left(a\right)\overset{\mathrm{def}}{=}1$ if $a={\mathrm{argmax}}_{a^{\prime }}u\left(d,a^{\prime }\right)$ (if there is more than one such a, we select one of them arbitrarily), and $\phi_{a}^{*}\left(d\right)\left(a\right)\overset{\mathrm{def}}{=}0$ otherwise. Then, for every δ∈DD and $\phi_{a}:D\to D\left(A\right)$, we have: $U\left(d^{*},\phi_{a}\left(d^{*}\right)\right)\leq U\left(d^{*},\phi_{a}^{*}\left(d^{*}\right)\right)\leq U\left(\delta ,\phi_{a}^{*}\right)$.

**Proof.**  Let $a^{*}$ be the action selected by $\phi_{a}^{*}\left(d^{*}\right)$, i.e., $\phi_{a}^{*}\left(d^{*}\right)\left(a^{*}\right)\overset{\mathrm{def}}{=}1$. Then, $u\left(d^{*},a^{*}\right)=\max_{a}u\left(d^{*},a\right)$. Let δ and $\phi_{a}$ be arbitrary elements of DD and D→D(A), respectively. Then:

$$\begin{array}{ccc} U(d^{*},\phi_{a}\left(d^{*}\right))= & \;\;\sum_{a\in A}\phi_{a}\left(d^{*}\right)\left(a\right)\;u\left(d^{*},a\right) & \\ \leq & \;\;\sum_{a\in A}\phi_{a}\left(d^{*}\right)\left(a\right)\;u\left(d^{*},a^{*}\right) & \left(\mathrm{since}\;u\left(d^{*},a^{*}\right)=\max_{a}u\left(d^{*},a\right)\right)\\ = & \;\;u(d^{*},a^{*}) & \left(\mathrm{since}\;\phi_{a}\left(d^{*}\right)\mathrm{is}\;a\;\mathrm{distribution}\right)\\ = & \;\;U(d^{*},\phi_{a}^{*}\left(d^{*}\right)) & \left(\mathrm{by}\;\mathrm{the}\;\mathrm{definition}\;\mathrm{of}a^{*}\right)\\ = & \;\;\sum_{d\in D}\delta \left(d\right)\;u\left(d^{*},\phi_{a}^{*}\left(d^{*}\right)\right) & \left(\mathrm{since}\;\delta \mathrm{is}\;a\;\mathrm{distribution}\right)\\ \leq & \;\;\sum_{d\in D}\delta \left(d\right)\;u\left(d,\phi_{a}^{*}\left(d\right)\right) & \left(\mathrm{by}\;\mathrm{the}\;\mathrm{definition}\;\mathrm{of}d^{*}\mathrm{and}\;\mathrm{of}\phi_{a}^{*}\right)\\ = & \;\;U(\delta ,\phi_{a}^{*}) & \end{array}$$

 ☐

As a consequence of Theorems 3 and 4, we can show that in the games, we consider that the payoff of the optimal mixed and behavioral strategy profiles coincide. Note that this result could also be derived from the result from standard game theory, which states that, in the cases we consider, for any behavioral strategy, there is a mixed strategy that yields the same payoff, and vice versa [15]. However, the proof of [15] relies on Khun’s theorem, which is non-constructive (and rather complicated, because it is for more general cases). In our scenario, the proof is very simple, as we will see in the following corollary. Furthermore, since such a result does not hold for leakage games with hidden choice, we think it will be useful to show the proof formally in order to analyze the difference.

**Corollary 2** (Equivalence of optimal strategies of types D(D→A) and D→D(A) in Game II)**.** Consider a defender-first sequential game with visible choice, and let $d^{*}$, $s_{a}^{*}$ and $\phi_{a}^{*}$ be defined as in Theorems 3 and 4, respectively. Then, $u\left(d^{*},s_{a}^{*}\left(d^{*}\right)\right)=U\left(d^{*},\phi_{a}^{*}\left(d^{*}\right)\right).$

**Proof.**  The result follows immediately by observing that $u\left(d^{*},s_{a}^{*}\left(d^{*}\right)\right)=\max_{a}u\left(d^{*},a\right)=u\left(d^{*},a^{*}\right)=U\left(d^{*},\phi_{a}^{*}\left(d^{*}\right)\right)$. ☐

#### 5.1.3. Game III (Attacker-First with Visible Choice)

This game is also a sequential game, but with the attacker as the leader. Therefore, it can be represented as a tuple of the form (A→D,A,u). It is the same as Game II, except that the roles of the attacker and the defender are inverted. In particular, the payoff of a mixed strategy profile $\left(\sigma_{d},\alpha \right)\in D\left(A\;\to \;D\right)\times DA$ is given by:

$$\begin{array}{c} U\left(\sigma_{d},\alpha \right)\overset{\mathrm{def}}{=}\underset{\begin{array}{c} s_{d}\leftarrow \sigma_{d}\\ a\leftarrow \alpha \end{array}}{E}u\left(s_{d}\left(a\right),a\right)=\sum_{\begin{array}{c} s_{d}:A\;\to \;D\\ a\in A \end{array}}\sigma_{d}\left(s_{d}\right)\;\alpha \left(a\right)\;u\left(s_{d}\left(a\right),a\right) \end{array}$$

and by Theorem 2 (2), the whole system can be equivalently regarded as channel $\underset{\begin{array}{c} s_{d}\leftarrow \sigma_{d}\\ a\leftarrow \alpha \end{array}}{\left\lfloor \;\cdot \;\right\rfloor }C_{s_{d}\left(a\right)a}$. For a behavioral strategy $\left(\phi_{d},\alpha \right)\in \left(A\to D\left(D\right)\right)\times DA$, the payoff is given by:

$$\begin{array}{c} U\left(\phi_{d},\alpha \right)\overset{\mathrm{def}}{=}\underset{a\leftarrow \alpha }{E}\;\;\underset{d\leftarrow \phi_{d}\left(a\right)}{E}u\left(d,a\right)=\sum_{a\in A}\alpha \left(a\right)\sum_{d\in D}\phi_{d}\left(a\right)\left(d\right)u\left(d,a\right) \end{array}$$

and by Theorem 2 (2), the whole system can be equivalently regarded as channel $\underset{a\leftarrow \alpha }{\left\lfloor \;\cdot \;\right\rfloor }\;\;\underset{d\leftarrow \phi_{d}\left(a\right)}{\left\lfloor \;\cdot \;\right\rfloor }C_{da}$.

Obviously, the same results that we have obtained in the previous section for Game II hold also for Game III, with the role of attacker and defender switched. We collect all these results in the following theorem.

**Theorem 5** (Pure-strategy Nash equilibria in Game III and equivalence of D(A→D) and (A→D(D)))**.** Consider a defender-first sequential game with visible choice. Let $a^{*}\overset{\mathrm{def}}{=}{\mathrm{argmax}}_{a}\min_{d}u\left(d,a\right)$. Let $s_{d}^{*}:A\;\to \;D$ be defined as $s_{d}^{*}\left(a\right)\overset{\mathrm{def}}{=}{\mathrm{argmin}}_{d}u\left(d,a\right)$, and let $\phi_{d}^{*}:A\to D\left(D\right)$ be defined as $\phi_{d}^{*}\left(a\right)\left(d\right)\overset{\mathrm{def}}{=}1$ if $d={\mathrm{argmin}}_{d^{\prime }}u\left(d^{\prime },a\right)$. Then:

*1*. For every α∈DA and $\sigma_{d}\in D\left(A\;\to \;D\right)$, we have $U\left(s_{d}^{*},\alpha \right)\leq u\left(s_{d}^{*}\left(a^{*}\right),a^{*}\right)\leq U\left(\sigma_{d},a^{*}\right)$.
*2*. For every α∈DA and $\phi_{d}:A\to D\left(D\right)$, we have: $U\left(\phi_{d}^{*},\alpha \right)\leq U\left(\phi_{d}^{*}\left(a^{*}\right),a^{*}\right)\leq U\left(\phi_{d}\left(a^{*}\right),a^{*}\right)$.
*3*. $u\left(s_{d}^{*}\left(a^{*}\right),a^{*}\right)=U\left(\phi_{d}^{*}\left(a^{*}\right),a^{*}\right)$.

**Proof.**  These results can be proven by following the same lines as the proofs of Theorems 3 and 4 and Corollary 2. ☐

**Example** **7.** Consider now the example of Section 3 in the setting of Game III, with uniform prior. If the attacker chooses zero, then the defender chooses zero, and the payoff is 12. If the attacker chooses one, then the defender chooses one, and the payoff is 23. The latter case is more convenient for the attacker; hence, the solution of the game is the strategy profile $\left(\delta^{*},\alpha^{*}\right)$ with $\delta^{*}\left(0\right)=0$, $\alpha^{*}\left(0\right)=0$.

#### 5.1.4. Game IV (Simultaneous with Hidden Choice)

The simultaneous game with hidden choice is a tuple (D,A,u). However, it is not an ordinary game in the sense that the payoff of a mixed strategy profile cannot be defined by averaging the payoff of the corresponding pure strategies. More precisely, the payoff of a mixed profile is defined by averaging on the strategy of the attacker, but not on that of the defender. In fact, when hidden choice is used, there is an additional level of uncertainty in the relation between the observables and the secret from the point of view of the attacker, since he/she is not sure about which channel is producing those observables. A mixed strategy δ for the defender produces a convex combination of channels (the channels associated with the pure strategies) with the same coefficients, and we know from previous sections that the vulnerability is a convex function of the channel and in general is not linear.

In order to define the payoff of a mixed strategy profile (δ,α), we need therefore to consider the channel that the attacker perceives given his/her limited knowledge. Let us assume that the action that the attacker draws from α is *a*. He does not know the action of the defender, but we can assume that he/she knows his/her strategy (each player can derive the optimal strategy of the opponent, under the assumption of common knowledge and rational players).

The channel the attacker will see is ${⨊}_{d\leftarrow \delta }C_{da}$, obtaining a corresponding payoff of $V\left[\pi ,{⨊}_{d\leftarrow \delta }C_{da}\right]$. By averaging on the strategy of the attacker, we obtain:

$$\begin{array}{c} U\left(\delta ,\alpha \right)\overset{\mathrm{def}}{=}\underset{\begin{array}{c} a\leftarrow \alpha \end{array}}{E}\;V\left[\pi ,\underset{d\leftarrow \delta }{⨊}C_{da}\right]=\sum_{a\in A}\;\alpha \left(a\right)\;V\left[\pi ,\underset{d\leftarrow \delta }{⨊}C_{da}\right]. \end{array}$$

From Theorem 2 (2), we derive: $U\left(\delta ,\alpha \right)=V\left[\pi ,{\left\lfloor \;\cdot \;\right\rfloor }_{a\leftarrow \alpha }\;{⨊}_{d\leftarrow \delta }C_{da}\right]$, and hence, the whole system can be equivalently regarded as channel ${\left\lfloor \;\cdot \;\right\rfloor }_{a\leftarrow \alpha }\;{⨊}_{d\leftarrow \delta }C_{da}$. Note that, by Proposition 4c, the order of the operators is interchangeable, and the system can be equivalently regarded as ${⨊}_{d\leftarrow \delta }\;{\left\lfloor \;\cdot \;\right\rfloor }_{a\leftarrow \alpha }C_{da}$. This shows the robustness of this model.

From Corollary 1, we derive that U(δ,α) is convex in δ and linear in η; hence, we can compute the Nash equilibrium by the minimax method.

**Example** **8.** *Consider now the example of Section 3 in the setting of Game IV. For δ∈DD and α∈DA, let p=δ(0) and q=α(0). The system can be represented by the channel $\left(C_{00}\;{}_{p}⊕\;C_{10}\right)\;{}_{q}\left\lfloor \;\cdot \;\right\rfloor \;\left(C_{01}\;{}_{p}⊕\;C_{11}\right)$ represented below.*

C00p⊕C10y=0y=1x=0pp¯x=110q⌊·⌋C01p⊕C11y=0y=1x=013+23p23−23px=123−23p13+23p

*For uniform π, we have Vπ,C00p⊕C10=1−12p, while $V\left[\pi ,C_{10}\;{}_{p}⊕\;C_{11}\right]$ is equal to 23−23p if p≤14 and equal to 13+23p if p>14. Hence the payoff, expressed in terms of p and q, is U(p,q)=q(1−12p)+q¯(23−23p) if p≤14 and U(p,q)=q(1−12p)+q¯(13+23p) if p>14. The Nash equilibrium can be computed by imposing that the partial derivatives of U(p,q) with respect to p and q are both zero, which means that we are in a saddle point. We have:*

$$\frac{\partial U(p,q)}{\partial q}=\left\{\begin{array}{cc} \frac{1}{3}+\frac{1}{6}\;p & ,\;if\;p\leq \frac{1}{4}\\ \frac{2}{3}-\frac{7}{6}\;p & ,\;if\;p>\frac{1}{4} \end{array}\right.\;\frac{\partial U(p,q)}{\partial p}=\left\{\begin{array}{cc} -\frac{2}{3}+\frac{1}{6}\;q & ,\;if\;p\leq \frac{1}{4}\\ \frac{2}{3}-\frac{7}{6}\;q & ,\;if\;p>\frac{1}{4} \end{array}\right.$$

We can see that the equations ∂U(p,q)∂q=0 and ∂U(p,q)∂p=0 do not have solutions in [0,1] for p≤14, while for p>14, they have solution p*=q*=47. The pair $\left(p^{*},q^{*}\right)$ thus constitutes the Nash equilibrium, and the corresponding payoff is U(p*,q*)=57.

#### 5.1.5. Game V (Defender-First with Hidden Choice)

This is a defender-first sequential game with imperfect information; hence, it can be represented as a tuple of the form $\left(D,\;K_{a}\to A,\;u\right)$, where $K_{a}$ is a partition of D. Since we are assuming perfect recall, and the attacker does not know anything about the action chosen by the defender in Phase 2, i.e., at the moment of the attack (except the probability distribution determined by his/her strategy), we must assume that the attacker does not know anything in Phase 1 either. Hence, the indistinguishability relation must be total, i.e., $K_{a}=\left\{D\right\}$. However, {D}→A is equivalent to A; hence, this kind of game is equivalent to Game IV. It is also a well-known fact in game theory that when in a sequential game the follower does not know the leader’s move before making his/her choice, the game is equivalent to a simultaneous game. (However, one could argue that, since the defender has already committed, the attacker does not need to perform the action corresponding to the Nash equilibrium, and any payoff-maximizing solution would be equally good for him.)

#### 5.1.6. Game VI (Attacker-First with Hidden Choice)

This game is also a sequential game with the attacker as the leader; hence, it is a tuple of the form (A→D,A,u). It is similar to Game III, except that the payoff is convex on the strategy of the defender, instead of linear. We will see, however, that this causes quite some deviation from the properties of Game III and from standard game theory.

The payoff of the mixed strategy profile $\left(\sigma_{d},\alpha \right)\in D\left(A\;\to \;D\right)\times DA$ is:

$$\begin{array}{c} U\left(\sigma_{d},\alpha \right)\overset{\mathrm{def}}{=}\underset{\begin{array}{c} a\leftarrow \alpha \end{array}}{E}\;V\left[\pi ,\underset{s_{d}\leftarrow \sigma_{d}}{⨊}C_{s_{d}\left(a\right)a}\right]=V\left[\pi ,\underset{a\leftarrow \alpha }{\left\lfloor \;\cdot \;\right\rfloor }\;\underset{s_{d}\leftarrow \sigma_{d}}{⨊}C_{s_{d}\left(a\right)a}\right], \end{array}$$

so the whole system can be equivalently regarded as channel $\underset{a\leftarrow \alpha }{\left\lfloor \;\cdot \;\right\rfloor }\;\underset{s_{d}\leftarrow \sigma_{d}}{⨊}C_{s_{d}\left(a\right)a}$.

The first important difference from Game III is that in Game VI, there may not exist optimal strategies, either mixed or behavioral, that are deterministic for the defender. On the other hand, for the attacker, there are always deterministic optimal strategies, and this is true independently of whether the defender uses mixed or behavioral strategies.

To show the existence of deterministic optimal strategies for the attacker, let us first introduce some standard notation for functions: given a variable *x* and an expression *M*, λx.M represents the function that on the argument *x* gives as a result the value of *M*. Given two sets *X* and *Y* where *Y* is provided with an ordering ≤, the point-wise ordering on X→Y is defined as follows: for f,g:X→Y, f≤g if and only if ∀x∈X.f(x)≤g(x).

**Theorem** **6** (Attacker’s pure-strategy Nash equilibrium in Game VI)**.** *Consider an attacker-first sequential game with hidden choice.*

*1*. Mixed strategies, type D(A→D). Let $a^{*}\overset{\mathrm{def}}{=}{\mathrm{argmax}}_{a}\min_{\sigma_{d}}V\left[\pi ,{⨊}_{s_{d}\leftarrow \sigma_{d}}C_{s_{d}\left(a\right)a}\right]$, and let $\sigma_{d}^{*}\overset{\mathrm{def}}{=}{\mathrm{argmin}}_{\sigma_{d}}\lambda a.V\left[\pi ,{⨊}_{s_{d}\leftarrow \sigma_{d}}C_{s_{d}\left(a\right)a}\right]$. Then, for every α∈DA and $\sigma_{d}\in D\left(A\;\to \;D\right)$ we have:
  $$U\left(\sigma_{d}^{*},\alpha \right)\leq U\left(\sigma_{d}^{*},a^{*}\right)\leq U\left(\sigma_{d},a^{*}\right)$$
*2*. Behavioral strategies, type A→D(D). Let $a^{*}\overset{\mathrm{def}}{=}{\mathrm{argmax}}_{a}\min_{\delta }V\left[\pi ,{⨊}_{d\leftarrow \delta }C_{da}\right]$, and let $\phi_{d}^{*}\overset{\mathrm{def}}{=}{\mathrm{argmin}}_{\phi_{d}}\lambda a.V\left[\pi ,{⨊}_{d\leftarrow \phi_{d}\left(a\right)}C_{da}\right]$ (the minimization is with respect to the point-wise ordering). Then, for every α∈DA and $\phi_{d}:A\to D\left(D\right)$, we have:
  $$U\left(\phi_{d}^{*},\alpha \right)\leq U\left(\phi_{d}^{*},a^{*}\right)\leq U\left(\phi_{d},a^{*}\right)$$

**Proof.** 

1. Let α and $\sigma_{d}$ be arbitrary elements of DA and D(A→D), respectively. Then:
  $$\begin{array}{ccc} U(\sigma_{d}^{*},\alpha )= & \;\;\sum_{a\in A}\alpha \left(a\right)\;V\left[\pi ,\underset{s_{d}\leftarrow \sigma_{d}^{*}}{⨊}C_{s_{d}\left(a\right)a}\right] & \\ \leq & \;\;\sum_{a\in A}\alpha \left(a\right)\;V\left[\pi ,\underset{s_{d}\leftarrow \sigma_{d}^{*}}{⨊}C_{s_{d}\left(a^{*}\right)a^{*}}\right] & \left(\mathrm{by}\;\mathrm{the}\;\mathrm{definition}\;\mathrm{of}a^{*}\mathrm{and}\sigma_{d}^{*}\right)\\ = & \;\;V\left[\pi ,\underset{s_{d}\leftarrow \sigma_{d}^{*}}{⨊}C_{s_{d}\left(a^{*}\right)a^{*}}\right]\;\left(\;=\;U\left(\sigma_{d}^{*},a^{*}\right)\right) & \left(\mathrm{since}\;\alpha \mathrm{is}\;a\;\mathrm{distribution}\right)\\ \leq & \;\;V\left[\pi ,\underset{s_{d}\leftarrow \sigma_{d}}{⨊}C_{s_{d}\left(a^{*}\right)a^{*}}\right] & \left(\mathrm{by}\;\mathrm{the}\;\mathrm{definition}\;\mathrm{of}\sigma_{d}^{*}\right)\\ = & \;\;U(\sigma_{d},a^{*}) & \end{array}$$
2. Let α and $\phi_{d}$ be arbitrary elements of DA and A→D(D), respectively. Then:
  $$\begin{array}{ccc} U(\phi_{d}^{*},\alpha )= & \;\;\sum_{a\in A}\alpha \left(a\right)\;V\left[\pi ,\underset{d\leftarrow \phi_{d}^{*}\left(a\right)}{⨊}C_{da}\right] & \\ \leq & \;\;\sum_{a\in A}\alpha \left(a\right)\;V\left[\pi ,\underset{d\leftarrow \phi_{d}^{*}\left(a^{*}\right)}{⨊}C_{da^{*}}\right] & \left(\mathrm{by}\;\mathrm{the}\;\mathrm{definition}\;\mathrm{of}a^{*}\mathrm{and}\phi_{d}^{*}\right)\\ = & \;\;V\left[\pi ,\underset{d\leftarrow \phi_{d}^{*}\left(a^{*}\right)}{⨊}C_{da^{*}}\right]\;\left(\;=\;U\left(\phi_{d}^{*},a^{*}\right)\right) & \left(\mathrm{since}\;\alpha \mathrm{is}\;a\;\mathrm{distribution}\right)\\ \leq & \;\;V\left[\pi ,\underset{d\leftarrow \phi_{d}\left(a^{*}\right)}{⨊}C_{da^{*}}\right] & \left(\mathrm{by}\;\mathrm{the}\;\mathrm{definition}\;\mathrm{of}\phi_{d}^{*}\right)\\ = & \;\;U(\phi_{d},a^{*}) & \end{array}$$

 ☐

We show now, with the following example, that the optimal strategies for the defender are necessarily probabilistic.

**Example** **9.** Consider the channel matrices $C_{ij}$ defined in Section 3 and define the following new channels: $C_{00}^{\prime }=C_{11}^{\prime }=C_{01}$ and $C_{10}^{\prime }=C_{01}^{\prime }=C_{10}$. Define $D_{p}$ as the result of the hidden choice, with probability p, between $C_{00}^{\prime }$ and $C_{10}^{\prime }$, i.e., $D_{p}\overset{\mathrm{def}}{=}C_{00}^{\prime }\;{}_{p}⊕\;C_{10}^{\prime }$, and observe that $D_{p}\left[0,0\right]=D_{p}\left[1,1\right]=p$ and $D_{p}\left[1,0\right]=D_{p}\left[0,1\right]=1-p$. Furthermore, $D_{p}=C_{01}^{\prime }\;{}_{1-p}⊕\;C_{11}^{\prime }$. The vulnerability of $D_{p}$, for uniform π, is Vπ,Dp=1−12p for p≤12 and $V\left[\pi ,D_{p}\right]=p$ for p>12; hence, independently of the choice of the attacker, the best strategy for the defender is to choose p=12. Every other value for p gives a strictly higher vulnerability. Therefore, the best mixed strategy for the defender is $\sigma_{d}^{*}$ defined as σd*(λa.0)=σd*(λa.1)=12. Similarly, the best behavioral strategy for the defender is $\phi_{d}^{*}$ defined as ϕd*(0)=ϕd*(1)=λd.12.

The second important difference from Game III is that in Game VI, behavioral strategies and mixed strategies are not necessarily equivalent. More precisely, there are cases in which the optimal strategy profile yields a different payoff depending on whether the defender adopts mixed strategies or behavioral ones. The following is an example in which this difference manifests itself.

**Example** **10.** *Consider again the example of Section 3, this time in the setting of Game VI, and still with uniform prior π. Let us analyze first the case in which the defender uses behavioral strategies.*

**Figure 2:**
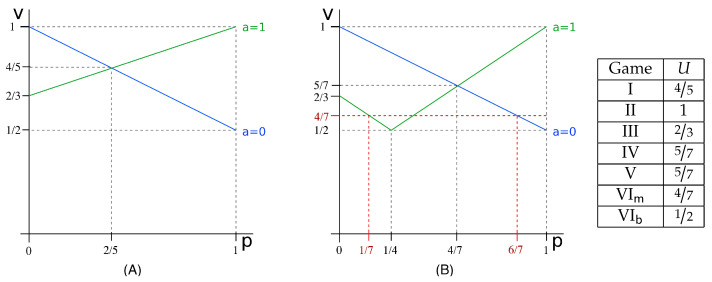
Summary of the results for the running example introduced in Section 3, for a uniform prior. Graph (**A**) is for the case of visible choice: it represents the Bayes vulnerability V of $C_{00}\;{}_{p}\left\lfloor \;\cdot \;\right\rfloor \;C_{10}$ and of $C_{01}\;{}_{p}\left\lfloor \;\cdot \;\right\rfloor \;C_{11}$ (cases a=0 and a=1, respectively), as a function of *p*; Graph (**B**) is for the case of hidden choice, and it represents the vulnerability of $C_{00}\;{}_{p}⊕\;C_{10}$ and of $C_{01}\;{}_{p}⊕\;C_{11}$ as a function of *p*. The table on the right gives the payoff in correspondence of the Nash equilibrium for the various games. VI${}_{m}$ and VI${}_{b}$ represent the attacker-first sequential games with defender strategy of type D(A→D) (mixed) and A→D(D) (behavioral), respectively.

*1*. Behavioral strategies, type A→D(D). If the attacker chooses zero, which corresponds to committing to the system $C_{00}\;{}_{p}⊕\;C_{10}$, then the defender will choose p=14, which minimizes its vulnerability. If he/she chooses one, which corresponds to committing to the system $C_{01}\;{}_{p}⊕\;C_{11}$, then the defender will choose p=1, which minimizes the vulnerability. In both cases, the leakage is p=12; hence, both of these strategies are solutions to the minimax. Note that in the first case, the strategy of the defender is probabilistic, while that of the attacker is pure in both cases.
*2*. Mixed strategies, type D(A→D). Observe that there are only four possible pure strategies for the defender, corresponding to the four functions $f_{ij}:A\;\to \;D$ for i,j∈{0,1} defined as $f_{ij}\left(a\right)\overset{\mathrm{def}}{=}i$ if i=j and $f_{ij}\left(a\right)\overset{\mathrm{def}}{=}a⊕i$ if i≠j. Consider a distribution $\sigma_{d}\in D\left(A\;\to \;D\right)$, and let $p_{ij}\overset{\mathrm{def}}{=}\sigma_{d}\left(f_{ij}\right)$. Then, we have $p_{ij}\geq 0$ and $\sum_{i,j}p_{ij}=1$. Observe that the attacker’s choice a=0 determines the matrix $C_{00}\;{}_{p}⊕\;C_{10}$, with $p=p_{00}+p_{10}$, whose vulnerability is Vπ,C00p⊕C10=1−12p. On the other hand, the attacker’s choice a=1 determines the matrix $C_{01}\;{}_{p^{\prime }}⊕\;C_{11}$, with $p^{\prime }=p_{00}+p_{01}$, whose vulnerability is Vπ,C01p′⊕C11=23−23p for p′≤14, and Vπ,C01p′⊕C11=13+23p for p′>14. By geometrical considerations (cf. the red dashed line in Figure 2), we can see that the optimal solutions for the defender are all those strategies, which give p=67 and p′=17, which yield payoff 47.

The fact that behavioral and mixed strategies are not equivalent is related to the non-existence of deterministic optimal strategies. In fact, it is easy to see that from a behavioral deterministic strategy, we can construct a (deterministic) mixed strategy, and vice versa.

Figure 2 illustrates the graphs of the vulnerability of the various channel compositions and summarizes the results of this section.

## 6. Comparing the Leakage Games

In previous section, we have computed the vulnerability for the running example in the various kinds of games introduced in Section 5. The values we have obtained, listed in decreasing order, are as follows: II:1;I:45;IV:57;V:57;III:23;VIm:47;VIb:12. This order is not accidental: in this section, we will prove that some of these relations between games hold for any vulnerability function, and for any prior. These results will allow us to reason about which kinds of scenarios and compositions are more convenient for the defender or, vice versa, for the attacker.

### 6.1. Simultaneous Games vs. Sequential Games

The relations between II, I and III and between IV–V and VI${}_{m}$ are typical in game theory: in any zero-sum sequential game, the leader’s payoff is less than or equal to his/her payoff in the corresponding simultaneous game. In fact, by acting first, the leader commits to an action, and this commitment can be exploited by the attacker to choose the best possible strategy relative to that action. (The fact that the leader has a disadvantage may seem counterintuitive because in many real games, it is the opposite: the player who moves first has an advantage. Such a discrepancy is due to the fact that these games feature preemptive moves, i.e., moves that, when made by one player, make impossible other moves for the other player. The games we are considering in this paper, on the contrary, do not consider preemptive moves.) In the following propositions, we give the precise formulation of these results in our framework, and we show how they can be derived formally.

**Proposition** **1** (Game II ⩾ Game I)**.** $$\begin{array}{cc} \min_{\delta }\max_{\sigma_{a}}V\left[\pi ,\underset{\begin{array}{c} d\leftarrow \delta \\ s_{a}\leftarrow \sigma_{a} \end{array}}{\left\lfloor \;\cdot \;\right\rfloor }C_{ds_{a}\left(d\right)}\right] & \geq \min_{\delta }\max_{\alpha }V\left[\pi ,\underset{\begin{array}{c} d\leftarrow \delta \\ a\leftarrow \alpha \end{array}}{\left\lfloor \;\cdot \;\right\rfloor }C_{da}\right] \end{array}$$

**Proof.**  We prove the first inequality as follows. Independently of δ, consider the attacker’s strategy $\sigma_{a}^{*}$ that assigns probability one to the function $s_{a}^{*}$ defined as $s_{a}^{*}\left(d\right)={\mathrm{argmax}}_{a}V\left[\pi ,C_{da}\right]$ for any d∈D. Then, we have that:

$$\begin{array}{ccc} \min_{\delta }\max_{\sigma_{a}}V\left[\pi ,\underset{\begin{array}{c} d\leftarrow \delta \\ s_{a}\leftarrow \sigma_{a} \end{array}}{\left\lfloor \;\cdot \;\right\rfloor }C_{ds_{a}\left(d\right)}\right]\geq & \;\;\min_{\delta }V\left[\pi ,\underset{\begin{array}{c} d\leftarrow \delta \\ s_{a}\leftarrow \sigma_{a}^{*} \end{array}}{\left\lfloor \;\cdot \;\right\rfloor }\;C_{ds_{a}\left(d\right)}\right] & \left(\mathrm{by}\;\mathrm{maximization}\;\mathrm{on}\;\sigma_{a}\right)\\ = & \;\;\min_{\delta }V\left[\pi ,\underset{d\leftarrow \delta }{\left\lfloor \;\cdot \;\right\rfloor }\;C_{ds_{a}^{*}\left(d\right)}\right] & \left(\mathrm{by}\;\mathrm{the}\;\mathrm{definition}\;\mathrm{of}\;\sigma_{a}^{*}\right)\\ = & \;\;\min_{\delta }\sum_{d}\delta \left(d\right)\;V\left[\pi ,C_{ds_{a}^{*}\left(d\right)}\right] & \left(\mathrm{by}\;\mathrm{Theorem}2\;\;(2)\right)\\ = & \;\;\min_{\delta }\sum_{d}\delta \left(d\right)\max_{\alpha }\sum_{a}\alpha \left(a\right)\;V\left[\pi ,C_{ds_{a}^{*}\left(d\right)}\right] & \left(\sin\mathrm{ce}\;\alpha \;\mathrm{is}\;a\;\mathrm{distribution}\right)\\ \geq & \;\;\min_{\delta }\sum_{d}\delta \left(d\right)\max_{\alpha }\sum_{a}\alpha \left(a\right)\;V\left[\pi ,C_{da}\right] & \left(\mathrm{by}\;\mathrm{the}\;\mathrm{definition}\;\mathrm{of}\;s_{a}^{*}\right)\\ \geq & \;\;\min_{\delta }\max_{\alpha }\sum_{d}\delta \left(d\right)\sum_{a}\alpha \left(a\right)\;V\left[\pi ,C_{da}\right] & \\ = & \;\;\min_{\delta }\max_{\alpha }V\left[\pi ,\underset{\begin{array}{c} d\leftarrow \delta \\ a\leftarrow \alpha \end{array}}{\left\lfloor \;\cdot \;\right\rfloor }C_{da}\right] & \left(\mathrm{by}\;\mathrm{Theorem}2\;\;(2)\right) \end{array}$$

 ☐

Note that the strategy $\sigma_{a}^{*}$ is optimal for the attacker, so the first of the above inequalities is actually an equality. It is easy to see that the second inequalities comprise an equality, as well, because of the maximization on α. Therefore, the only inequalities that may be strict are comprised by the third one, and the reason why it may be strict is that on the left-hand side, α depends on *d* (and on δ), while on the right-hand side, α depends on δ, but not the actual *d* (that will be sampled from δ). This corresponds to the fact that in the defender-first sequential game, the attacker chooses his/her strategy after he/she knows the action *d* chosen by the defender, while in the simultaneous game, the attacker knows the strategy of the defender (i.e., the distribution δ he/she will use to choose probabilistically his/her actions), but not the actual action *d* that the defender will choose.

Analogous considerations can be done for the simultaneous versus the attacker-first case, which we will examine next.

**Proposition** **2** (Game I ≥ Game III)**.** $$\begin{array}{c} \min_{\delta }\max_{\alpha }V\left[\pi ,\underset{\begin{array}{c} d\leftarrow \delta \\ a\leftarrow \alpha \end{array}}{\left\lfloor \;\cdot \;\right\rfloor }C_{da}\right]\;\;\geq \;\;\max_{\alpha }\min_{\sigma_{d}}V\left[\pi ,\underset{\begin{array}{c} s_{d}\leftarrow \sigma_{d}\\ a\leftarrow \alpha \end{array}}{\left\lfloor \;\cdot \;\right\rfloor }C_{s_{d}\left(a\right)a}\right] \end{array}$$

**Proof.**  Independently of α, consider the defender’s strategy $\sigma_{d}^{*}$ that assigns probability one to the function $s_{d}^{*}$ defined as $s_{d}^{*}\left(a\right)={\mathrm{argmin}}_{d}V\left[\pi ,C_{da}\right]$ for any a∈A. Then, we have that:

$$\begin{array}{ccc} \min_{\delta }\max_{\alpha }V\left[\pi ,\underset{\begin{array}{c} d\leftarrow \delta \\ a\leftarrow \alpha \end{array}}{\left\lfloor \;\cdot \;\right\rfloor }C_{da}\right]= & \;\;\min_{\delta }\max_{\alpha }\sum_{d}\delta \left(d\right)\sum_{a}\alpha \left(a\right)\;V\left[\pi ,C_{da}\right] & \left(\mathrm{by}\;\mathrm{Theorem}\;2\;(2)\right)\\ = & \;\;\max_{\alpha }\min_{\delta }\sum_{d}\delta \left(d\right)\sum_{a}\alpha \left(a\right)\;V\left[\pi ,C_{da}\right] & \left(\mathrm{by}\;\mathrm{Theorem}\;1\right)\\ \geq & \;\;\max_{\alpha }\sum_{a}\alpha \left(a\right)\min_{d}\;V\left[\pi ,C_{da}\right] & \\ = & \;\;\max_{\alpha }\sum_{a}\alpha \left(a\right)\;V\left[\pi ,C_{s_{d}^{*}\left(a\right)a}\right] & \left(\mathrm{by}\;\mathrm{the}\;\mathrm{definition}\;\mathrm{of}\;s_{d}^{*}\right)\\ = & \;\;\max_{\alpha }\sum_{a}\alpha \left(a\right)\sum_{s_{d}}\sigma_{d}^{*}\left(s_{d}\right)\;V\left[\pi ,C_{s_{d}\left(a\right)a}\right] & \left(\mathrm{by}\;\mathrm{the}\;\mathrm{definition}\;\mathrm{of}\;\sigma_{d}^{*}\right)\\ = & \;\;\max_{\alpha }V\left[\pi ,\underset{\begin{array}{c} s_{d}\leftarrow \sigma_{d}^{*}\\ a\leftarrow \alpha \end{array}}{\left\lfloor \;\cdot \;\right\rfloor }C_{s_{d}\left(a\right)a}\right] & \left(\mathrm{by}\;\mathrm{Theorem}\;2\;(2)\right)\\ \geq & \;\;\max_{\alpha }\min_{\sigma_{d}}V\left[\pi ,\underset{\begin{array}{c} s_{d}\leftarrow \sigma_{d}\\ a\leftarrow \alpha \end{array}}{\left\lfloor \;\cdot \;\right\rfloor }C_{s_{d}\left(a\right)a}\right] & \left(\mathrm{by}\;\mathrm{minimization}\;\mathrm{on}\;\sigma_{d}\right) \end{array}$$

 ☐

Again, the strategy $\sigma_{d}^{*}$ is optimal for the attacker, so the last of the above inequalities is actually an equality. Therefore, the only inequality that may be strict is the first one, and the strictness is due to the fact that on the left-hand side, δ does not depend on *a*, while on the right-hand side, it does. Intuitively, this corresponds to the intuition that if the defender knows the action of the attacker, then it may be able to choose a better strategy to reduce the leakage.

**Proposition** **3** (Game IV ⩾ Game VI${}_{m}$)**.** $$\begin{array}{cc} \min_{\delta }\max_{\alpha }V\left[\pi ,\underset{a\leftarrow \alpha }{\left\lfloor \;\cdot \;\right\rfloor }\;\underset{d\leftarrow \delta }{⨊}C_{da}\right] & \geq \max_{\alpha }\min_{\sigma_{d}}V\left[\pi ,\underset{a\leftarrow \alpha }{\left\lfloor \;\cdot \;\right\rfloor }\;\underset{s_{d}\leftarrow \sigma_{d}}{⨊}C_{s_{d}\left(a\right)a}\right] \end{array}$$

**Proof.**  Given α∈DA, let $\delta_{\alpha }^{*}\overset{\mathrm{def}}{=}\min_{\delta }\sum_{a}\alpha \left(a\right)V\left[\pi ,{⨊}_{d\leftarrow \delta }C_{da}\right]$. For any d∈D, let $f_{d}$ be the constant function defined as $f_{d}\left(a\right)=d$ for any a∈A, and define $\sigma_{d}^{*}\in D\left(A\;\to \;D\right)$ as $\sigma_{d}^{*}\left(f_{d}\right)\overset{\mathrm{def}}{=}\delta_{\alpha }^{*}\left(d\right)$ for any d∈D. Let $U\left(\delta ,\alpha \right)=V\left[\pi ,{\left\lfloor \;\cdot \;\right\rfloor }_{a\leftarrow \alpha }\;{⨊}_{d\leftarrow \delta }C_{da}\right]$. Then, U(δ,α) is convex in δ and linear in α. Hence, we have:

$$\begin{array}{ccc} \min_{\delta }\max_{\alpha }V\left[\pi ,\underset{a\leftarrow \alpha }{\left\lfloor \;\cdot \;\right\rfloor }\;\underset{d\leftarrow \delta }{⨊}C_{da}\right]= & \;\;\max_{\alpha }\min_{\delta }V\left[\pi ,\underset{a\leftarrow \alpha }{\left\lfloor \;\cdot \;\right\rfloor }\;\underset{d\leftarrow \delta }{⨊}C_{da}\right] & \left(\mathrm{by}\;\mathrm{Theorem}\;1\right)\\ = & \;\;\max_{\alpha }V\left[\pi ,\underset{a\leftarrow \alpha }{\left\lfloor \;\cdot \;\right\rfloor }\;\underset{d\leftarrow \delta_{\alpha }^{*}}{⨊}C_{da}\right] & \left(\mathrm{by}\;\mathrm{the}\;\mathrm{definition}\;\mathrm{of}\;\delta_{\alpha }^{*}\right)\\ = & \;\;\max_{\alpha }V\left[\pi ,\underset{a\leftarrow \alpha }{\left\lfloor \;\cdot \;\right\rfloor }\;\underset{f_{d}\leftarrow \sigma_{d}^{*}}{⨊}C_{f_{d}\left(a\right)a}\right] & \left(\mathrm{by}\;\mathrm{the}\;\mathrm{definition}\;\mathrm{of}\;\sigma_{d}^{*}\right)\\ \geq & \;\;\max_{\alpha }\min_{\sigma_{d}}V\left[\pi ,\underset{a\leftarrow \alpha }{\left\lfloor \;\cdot \;\right\rfloor }\;\underset{s_{d}\leftarrow \sigma_{d}}{⨊}C_{s_{d}\left(a\right)a}\right] & \left(\mathrm{by}\;\mathrm{minimization}\;\mathrm{on}\;\sigma_{d}\right) \end{array}$$

 ☐

### 6.2. Visible Choice vs. Hidden Choice

We consider now the case of Games III and IV–V. In the running example, the payoff for III is lower than for IV–V, but it is easy to find other cases in which the situation is reversed. For instance, if in the running example, we set $C_{11}$ to be the same as $C_{01}$, the payoff for III will be 1 (corresponding to the choice a=1 for the attacker), and that for IV–V will be 23 (corresponding to the Nash equilibrium p*=q*=23. Therefore, we conclude that Games III and IV–V are incomparable: there is no general ordering between them.

The relation between Games I and IV comes from the fact that they are both simultaneous games, and the only difference is the way in which the payoff is defined. The same holds for the case of Games III and VI${}_{m}$, which are both attacker-first sequential games. The essence of the proof is expressed by the following proposition.

**Proposition** **4** (Visible choice ⩾ hidden choice)**.** For every a∈A and every δ∈DD, we have:   $V\left[\pi ,{\left\lfloor \;\cdot \;\right\rfloor }_{d\leftarrow \delta }C_{da}\right]\;\;\geq \;\;V\left[\pi ,{⨊}_{d\leftarrow \delta }C_{da}\right].$

**Proof.**  $$\begin{array}{ccc} V\left[\pi ,\underset{d\leftarrow \delta }{\left\lfloor \;\cdot \;\right\rfloor }C_{da}\right]\;\;= & \;\;\sum_{d\in D}\delta \left(d\right)\;V\left[\pi ,C_{da}\right] & \left(\mathrm{by}\;\mathrm{Theorem}\;2\;(2)\right)\\ \geq & \;\;\;V\left[\pi ,\underset{d\leftarrow \delta }{⨊}C_{da}\right] & \left(\mathrm{by}\;\mathrm{Theorem}\;2\;(1)\right) \end{array}$$

 ☐

From the above proposition, we can derive immediately the following corollaries:

**Corollary** **3** (Game I ⩾ Game IV)**.** $$\begin{array}{c} \min_{\delta }\max_{\alpha }V\left[\pi ,\underset{\begin{array}{c} d\leftarrow \delta \\ a\leftarrow \alpha \end{array}}{\left\lfloor \;\cdot \;\right\rfloor }C_{da}\right]\;\;\geq \;\;\min_{\delta }\max_{\alpha }V\left[\pi ,\underset{a\leftarrow \alpha }{\left\lfloor \;\cdot \;\right\rfloor }\;\underset{d\leftarrow \delta }{⨊}C_{da}\right] \end{array}$$

**Corollary** **4** (Game III ⩾ Game VI${}_{m}$)**.** $$\begin{array}{c} \max_{\alpha }\min_{\sigma_{d}}V\left[\pi ,\underset{\begin{array}{c} s_{d}\leftarrow \sigma_{d}\\ a\leftarrow \alpha \end{array}}{\left\lfloor \;\cdot \;\right\rfloor }C_{s_{d}\left(a\right)a}\right]\;\;\geq \;\;\max_{\alpha }\min_{\sigma_{d}}V\left[\pi ,\underset{a\leftarrow \alpha }{\left\lfloor \;\cdot \;\right\rfloor }\;\underset{s_{d}\leftarrow \sigma_{d}}{⨊}C_{s_{d}\left(a\right)a}\right] \end{array}$$

Finally, we show that the vulnerability for the optimal solution in Game VI${}_{m}$ is always greater than or equal to that of Game VI${}_{b}$, which means that for the defender, it is always convenient to use behavioral strategies. We can state actually a more general result: for any mixed strategy, there is always a behavioral strategy that gives the same payoff.

**Proposition** **5.** *For any α∈DA and any $\sigma_{d}\in D\left(A\;\to \;D\right)$, there exists $\phi_{d}:A\to D\left(D\right)$ such that:*

$$V\left[\pi ,\underset{a\leftarrow \alpha }{\left\lfloor \;\cdot \;\right\rfloor }\;\underset{d\leftarrow \phi_{d}\left(a\right)}{⨊}C_{da}\right]\;=\;V\left[\pi ,\underset{a\leftarrow \alpha }{\left\lfloor \;\cdot \;\right\rfloor }\;\underset{s_{d}\leftarrow \sigma_{d}}{⨊}C_{s_{d}\left(a\right)a}\right]$$

**Proof.**  For $\sigma_{d}\in D\left(A\;\to \;D\right)$, define $\phi_{d}:A\to D\left(D\right)$ as follows: for any a∈A and d∈D,

$$\phi_{d}\left(a\right)\left(d\right)\overset{\mathrm{def}}{=}\sum_{s_{d}\left(a\right)=d}\sigma_{d}\left(s_{d}\right)$$

and observe that for every a∈A, we have $\underset{d\leftarrow \phi_{d}\left(a\right)}{⨊}C_{da}=\underset{s_{d}\leftarrow \sigma_{d}}{⨊}C_{s_{d}\left(a\right)a}$. ☐

From this proposition, we derive immediately the following corollary:

**Corollary** **5.** $$\max_{\alpha }\min_{\sigma_{d}}V\left[\pi ,\underset{a\leftarrow \alpha }{\left\lfloor \;\cdot \;\right\rfloor }\;\underset{s_{d}\leftarrow \sigma_{d}}{⨊}C_{s_{d}\left(a\right)a}\right]\;\geq \;\max_{\alpha }\min_{\phi_{d}}V\left[\pi ,\underset{a\leftarrow \alpha }{\left\lfloor \;\cdot \;\right\rfloor }\;\underset{d\leftarrow \phi_{d}\left(a\right)}{⨊}C_{da}\right]$$

**Figure 3:**
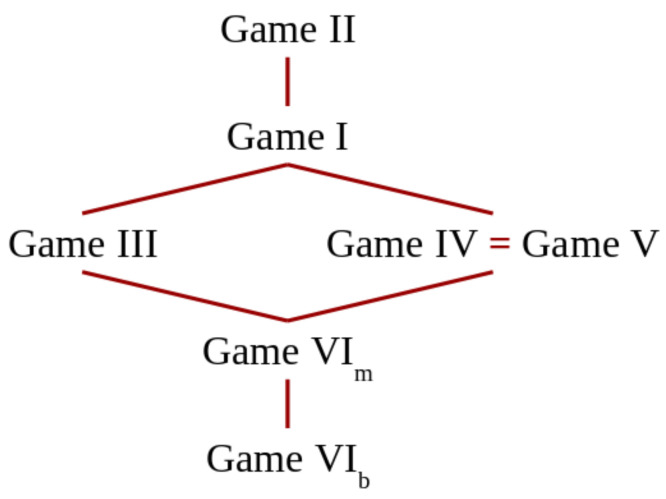
Order of games w.r.t. the payoff in the Nash equilibrium. Games higher in the lattice have larger payoff.

The lattice in Figure 3 illustrates the results of this section about the relations between the various games. These relations can be used by the defender as guidelines to better protect the system, if he/she has some control over the rules of the game. Obviously, for the defender, the games lower in the ordering are to be preferred to compose protocols, since they yield a lower vulnerability for the result.

## 7. Case Study: A Safer, Faster Password-Checker

In this section, we apply our game-theoretic, compositional approach to show how a defender can mitigate an attacker’s typical timing side-channel attack, while avoiding the usual burden imposed on the password-checker’s efficiency by measures that make time consumption constant.

The following sections are organized as follows: We first provide a formalization of the trade-off between efficiency and security in password checkers using our framework of leakage games. We then illustrate the approach in a simple instance of the program for 3-bit passwords. Finally, we provide general results for the *n*-bit case regarding the defender’s optimal strategy in equilibrium.

### 7.1. Modeling the Trade-Off between Efficiency and Security as a Game

Consider the password-checker PWD_1…n_ of Algorithm 1, which performs a bitwise-check of an *n*-bit low-input $a=a_{1},a_{2},\ldots ,a_{n}$ provided by the attacker against an *n*-bit secret password $x=x_{1},x_{2},\ldots ,x_{n}$. The bits are compared in increasing order (1, 2, …, *n*), with the low-input being rejected as soon as it mismatches the secret, and accepted otherwise.

**Algorithm 1:** Password-checker PWD_1…n_.
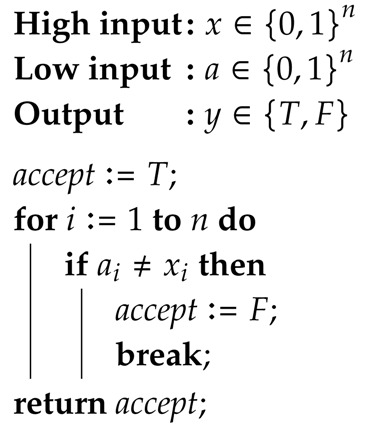


The attacker can choose low-inputs to try to gain information about the password. Obviously, in case PWD_1…n_ accepts the low-input, the attacker learns that the password value is a=x. Yet, even when the low-input is rejected, there is some leakage of information: from the duration of the execution, the attacker can estimate how many iterations have been performed before the low-input was rejected, thus inferring a prefix of the secret password.

To model this scenario, let $X={\left\{0,1\right\}}^{n}$ be the set of all possible *n*-bit passwords and Y={F}×{1,2,…,n}∪{T}×{n}={(F,1),(F,2),(F,3),…,(F,n),(T,n)} be the set of observables produced by the system. Each observable is an ordered pair whose first element indicates whether or not the password was accepted (*T* or *F*, respectively), and the second element indicates the duration of the computation (1, 2, …, or *n* iterations).

For instance, consider a scenario with 3-bit passwords. Let PWD_123_ be a password checker that performs the bitwise comparison in increasing order (1, 2, 3). Channel $C_{123,101}$ in Table 4 models PWD_123_’s behavior when the attacker provides low-input a=101. Note that this channel represents the fact that PWD_123_ accepts the low-input when the secret is x=101 (the channel outputs (T,3) with probability one), and otherwise rejects the low-input in a certain number of steps (e.g., the checker rejects the low-input in two steps when the password is x=110, so in this case, the channel outputs (F,2) with probability one).

To quantify the password checker’s leakage of information, we will adopt Bayes vulnerability, so the prior Bayes vulnerability $V\left[\pi \right]$ will correspond to the probability that the attacker guesses correctly the password in one try, whereas the posterior Bayes vulnerability $V\left[\pi ,C\right]$ will correspond to the probability that the attacker guesses correctly the password in one try, after he/she observes the output of the channel (i.e., after he/she has measured the time needed for the checker to accept or reject the low-input). For instance, in the 3-bit password scenario, if the prior distribution on all possible 3-bit passwords is $\hat{\pi }=\left(0.0137,0.0548,0.2191,0.4382,0.0002,0.0002,0.0548,0.2191\right)$, the corresponding prior Bayes vulnerability is $V\left[\hat{\pi }\right]=0.4382$. For prior $\hat{\pi }$ above, the posterior Bayes vulnerability of channel $C_{123,101}$ is $V\left[\hat{\pi },C_{123,101}\right]=0.6577$, which represents an increase in Bayes vulnerability of about 50%).

A way to mitigate this timing side-channel is to make the checker’s execution time independent of the secret. This can be done by by eliminating the break command within the loop in PWD_1…n_, so no matter when the matching among high and low input happens, the password checker will always need *n* iterations to complete. For instance, in the context of our 3-bit password example, we can let PWD_cons_ be a constant-time 3-bit password checker that applies this counter measure. Channel $C_{\mathrm{cons},101}$ from Table 5 models PWD_cons_’s behavior when the attacker’s low-input is a=101. Note that this channel reveals only whether or not the low-input matches the secret value, but does not allow the attacker to infer a prefix of the password. Indeed, this channel’s posterior Bayes vulnerability is $V\left[\hat{\pi },C_{123,101}\right]=0.4384$, which brings the multiplicative Bayes leakage down to an increase of only about 0.05%.

However, the original program is substantially more efficient than the modified one. Consider the general case of *n*-bit passwords and assume that either the password, or the program’s low input, is chosen uniformly at random. Because of this assumption, each bit being checked in the original program has probability 12 of being rejected. Hence, the program will finish after one iteration with probability 12, after two iterations with probability 14, and so on, up to the *n*-th iteration. After that, the program always finishes, so with the remaining probability $2^{-n}$, the program finishes after *n* iterations, giving a total expected time of:

$$\sum_{k=1}^{n}k2^{-k}+n2^{-n}=2\left(1-2^{-n}\right)\;.$$

The above derivation is based on the series $\sum_{k=1}^{n}kz^{k}=z\frac{1-\left(n+1\right)z^{n}+nz^{n+1}}{{\left(1-z\right)}^{2}}$. Hence, the expected running time of the original program (under the uniform assumption) is constant: always bounded by two, and converging to two as *n* grows. On the other hand, the running time of the modified constant-time program is *n* iterations, an n2-fold increase.

Seeking some compromise between security and efficiency, assume the defender can employ different versions of the password-checker, each performing the bitwise comparison among low-input *a* and secret password *x* in a different order. More precisely, there is one version of the checker for every possible order in which the index *i* ranges in the control of the loop in Algorithm 1.

To determine a defender’s best choice of which versions of the checker to run, we model this problem as a game. The attacker’s set of actions A consists of all possible $2^{n}$ low-inputs to the checker, and the defender’s set of actions D consists of all n! orders in which the checker can perform the bitwise comparison. There is, then, a channel $C_{ad}:X\times Y\to R$ for each possible combination of d∈D, a∈A. In our framework, the payoff of a mixed strategy profile (δ,α) is given by: $U\left(\delta ,\alpha \right)=E_{\begin{array}{c} a\leftarrow \alpha \end{array}}\;V\left[\pi ,{⨊}_{d\leftarrow \delta }C_{da}\right].$ For each pure strategy profile (d,a), the payoff of the game will be the posterior Bayes vulnerability of the resulting channel $C_{da}$ (since, if we are measuring the information leakage, the prior vulnerability is the same for every channel once the prior is fixed).

In the 3-bit password scenario, the attacker’s actions A={000,001,010,011,100,101,110,111} are all possible 3-bit low-inputs, and the defender’s D={123,132,213,231,312,321} are all possible versions of the password checker (each action represents the order in which the 3 bits are checked). Table 6 depicts the corresponding payoffs of all 48 possible resulting channels $C_{ad}$ with d∈D, a∈A, when the prior is still $\hat{\pi }=\left(0.0137,0.0548,0.2191,0.4382,0.0002,0.0002,0.0548,0.2191\right)$. Note that the attacker’s and defender’s actions substantially affect the effectiveness of the attack: vulnerability ranges between 0.4934 and 0.9311 (and so, multiplicative leakage is in the range between an increase of 12% and one of 112%). Using techniques from [6], we can compute the best (mixed) strategy for the defender in this game, which turns out to be:

$$\begin{array}{c} \delta^{*}=\left(0.1667,0.1667,0.1667,0.1667,0.1667,0.1667\right). \end{array}$$

This strategy is part of an equilibrium and guarantees that for any choice of the attacker, the posterior Bayes vulnerability is at most 0.6573 (so the multiplicative leakage is bounded by 50%, an intermediate value between the minimum of about 12% and the maximum of about 112%).

The running time, on the other hand, of this new password-checker is the same as that of the original one. Under the assumption that either the password or the low-input is uniformly distributed, each check fails with probability 12, giving a total expected time of $2(1-2^{-n})$. Hence, this technique substantially decreases the program’s information leakage, without affecting at all its expected running time.

### 7.2. On Optimal Strategies for the Defender

Interestingly, in the 3-bit password case study from the previous section, the defender’s optimal strategy consists of uniformly sampling among all available versions of the checker. A uniform distribution seems to be an adequate candidate for the defender, but is it always the best choice for any prior and any number of bits?

We first answer this question in the case of a uniform prior π for arbitrary *n*-bit passwords, which already turns out to be challenging. Under this prior, and exploiting a crucial symmetry of the password checker (see the proof of Theorem 7), we can show that all strategies for the adversary are in fact equivalent, namely:

$$U\left(\alpha ,\delta \right)=U\left(\alpha^{\prime },\delta \right)\;\mathrm{for}\;\mathrm{all}\;\alpha ,\alpha^{\prime },\delta \;.$$

For the defender, on the other hand, the situation is far from trivial: although all pure strategies *d* are still equivalent, U(α,δ) does in general depend on δ. By exploiting another symmetry of the password checker together with the symmetry of V, we can show that a uniform strategy is indeed optimal for the defender, as stated in the following result.

**Theorem** **7.** Consider the password checker program of Algorithm 1 for *n*-bit passwords, where the attacker controls the low input to the checker and the defender controls the order in which the bits are checked. If the prior π on possible passwords is uniform and the payoff is given by the posterior Bayes-vulnerability: $U\left(\delta ,\alpha \right)=E_{\begin{array}{c} a\leftarrow \alpha \end{array}}\;V\left[\pi ,{⨊}_{d\leftarrow \delta }C_{da}\right]$, then the strategy $\left(\delta^{*},\alpha \right)$ where $\delta^{*}$ is uniform and α is arbitrary is an equilibrium strategy.

Perhaps surprisingly, however, Theorem 7 does not generalize to non-uniform priors (or to different vulnerability metrics). More precisely, when the prior on passwords is not uniform, the defender may benefit from assigning different probabilities to different versions of the password checker. This subtlety arises from the fact that the defender’s goal is not to maximize the attacker’s uncertainty about the selected password checker itself (i.e., the defender’s action), but it is rather to maximize the attacker’s uncertainty about the secret value. The following examples illustrate this (perhaps counter-intuitive) fact.

Consider again a 3-bit password scenario, similar to that of the previous section. Assume that the attacker knows only that the first bit of the password is surely zero, so that the prior on secrets is:

$$\pi^{\left(A\right)}=\left(0.25,\;0.25,\;0.25,\;0.25,\;0,\;0,\;0,\;0\right).$$

The payoff table for this case is presented in Table 7, and a corresponding equilibrium defender’s best strategy, computed using techniques from [6], is:

$$\delta^{*(A)}=\left(0.25,0.25,0,0.25,0,0.25\right).$$

Note that this equilibrium means that the defender never has to check the bits in the order (2, 1, 3) or in the order (3, 1, 2). The game’s payoff (i.e., posterior Bayes vulnerability) in this case is 0.5625, which is smaller than the payoff of 0.5833 that would ensue in case the defender’s strategy were uniform. This means that, from the point of view of the defender, uniformly randomizing is not optimal.

Now, assume that the attacker knows that some passwords are more likely than others, even if all are possible, as reflected in the prior:

$$\pi^{\left(B\right)}=\left(0.25,\;0.20,\;0.15,\;0.10,\;0.10,\;0.10,\;0.05,\;0.05\right).$$

The payoff table for this case is presented in Table 8, and a corresponding equilibrium defender’s best strategy can be computed to be:

$$\delta^{*(B)}=\left(0.1974,\;0.1974,\;0.1316,\;0.1316,\;0.1711,\;0.1711\right).$$

Note that this equilibrium means that every version of the checker may be selected by the defender, but the probability distribution is not uniform. The game’s payoff (i.e., posterior Bayes vulnerability) in this case is 0.4553, which is again smaller than the payoff of 0.4666 that would ensue in case the defender’s strategy were uniform.

## 8. Related Work

Many studies have applied game theory to analyses of security and privacy in networks [18,19,20], cryptography [21], anonymity [22], location privacy [23] and intrusion detection [24], to cite a few. See [25] for a survey.

In the context of quantitative information flow, most works consider only passive attackers. Boreale and Pampaloni [4] considered adaptive attackers, but not adaptive defenders, and show that in this case, the attacker’s optimal strategy can be always deterministic. Mardziel et al. [5] proposed a model for both adaptive attackers and defenders, but in none of their extensive case-studies did the attacker need a probabilistic strategy to maximize leakage. In this paper, we characterize when randomization is necessary, for either attacker or defender, to achieve optimality in our general information leakage games.

Security games have been employed to model and analyze payoffs between interacting agents, especially between a defender and an attacker. Korzhyk et al. [26] theoretically analyzed security games and studied the relationships between Stackelberg and Nash equilibria under various forms of imperfect information. Khouzani and Malacaria [27] studied leakage properties when perfect secrecy was not achievable due to constraints on the allowable size of the conflating sets and provided universally optimal strategies for a wide class of entropy measures and for *g*-entropies. In particular, they prove that designing a channel with minimum leakage is equivalent to computing Nash equilibria in a corresponding two-player zero-sum game of incomplete information for a range of entropy measures. These works, contrary to ours, do not consider games with hidden choice, in which optimal strategies differ from traditional game-theory.

Several security games have modeled leakage when the sensitive information is the defender’s choices themselves, rather than a system’s high input. For instance, Alon et al. [28] propose zero-sum games in which a defender chooses probabilities of secrets and an attacker chooses and learns some of the defender’s secrets. Then, they present how the leakage of the defender’s secrets has an influence on the defender’s optimal strategy. More recently, Xu et al. [29] showed zero-sum games in which the attacker obtains partial knowledge on the security resources that the defender protects and provided the defender’s optimal strategy under the attacker’s knowledge. Contrary to these studies, in this paper, we assume that a secret value is drawn from some prior distribution and is not the defender’s strategy itself.

Security games have also been used to provide optimal trade-offs between two conflicting desirable properties. Khouzani et al. [30] studied the clash between security and usability in the password selection and presented a game-theoretic framework for determining an optimal trade-off. They analyzed guessing attacks and derived the optimal policies for secret picking as Nash/Stackelberg equilibria. Yang et al. [31] proposed a game-theoretic framework to analyze user behavior in anonymity networks. They considered the cost of anonymity in terms of the loss of utility. They also considered incentives and their impact on users’ cooperation. Shokri et al. [32] presented a game-theoretic model for a designer to find the optimal privacy mechanism by taking the adversary’s knowledge into account. More specifically, they showed a Stackelberg Bayesian game in which a user first chooses a location obfuscation mechanism to maximize his/her location privacy and then an adversary tries to estimate the user’s location to minimize its error. In contrast, our work presents a more general framework that is not limited to a particular domain and focuses on protocol composition as a method to limit the leakage.

Regarding channel operators, the sequential and parallel composition of channels have been studied (e.g., [33]), but we are unaware of any explicit definition and investigation of hidden and visible choice operators. Although Kawamoto et al. [34] implicitly used the hidden choice to model a probabilistic system as the weighted sum of systems, they did not derive the set of algebraic properties we do for this operator, and for its interaction with the visible choice operator.

## 9. Conclusions and Future Work

In this paper, we used protocol composition to model the introduction of noise performed by the defender to prevent leakage of sensitive information. More precisely, we formalized visible and hidden probabilistic choices of different protocols. We then formalized the interplay between defender and attacker in a game-theoretic framework adapted to the specific issues of QIF, where the payoff is information leakage. We considered various kinds of leakage games, depending on whether players act simultaneously or sequentially, and whether the choices of the defender are visible or not to the attacker. We established a hierarchy of these games and provided methods for finding the optimal strategies (at the points of equilibrium) in the various cases. We also proved that in a sequential game with hidden choice, the behavioral strategies are more advantageous for the defender than the mixed strategies. This contrasts with the standard game theory, where the two types of strategies are equivalent.

As future research, we would like to extend leakage games to the case of repeated observations, i.e., when the attacker can observe the outcomes of the system in successive runs, under the assumption that both attacker and defender may change the channel in each run. We would also like to extend our framework to non zero-sum games, in which the costs of attack and defense are not equivalent, and to analyze differentially-private mechanisms. 

## Figures and Tables

**Table 1:**
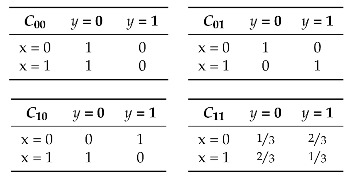
The four channels $C_{da}$ for d,a∈{0,1} for the running example.

**Table 2:** Bayes vulnerability of each channel $C_{da}$ for the running example.

V	*a* = 0	*a* = 1
d = 0	12	1
d = 1	1	23

**Table 3:** Kinds of games we consider. Sequential games have perfect information, except for Game V.

		Order of Action		
		Simultaneous	Defender First	Attacker First
Defender’s choice	visible ⌊·⌋	Game I	Game II	Game III
	hidden ⨊	Game IV	Game V	Game VI

**Table 4:** Channel $C_{123,101}$ modeling the case in which the defender compares bits in order (1, 2, 3) and the attacker picks low-input 101.

$C_{123,101}$	*y* = (*F*, 1)	*y* = (*F*, 2)	*y* = (*F*, 3)	*y* = (*T*, 3)
x = 000	1	0	0	0
x = 001	1	0	0	0
x = 010	1	0	0	0
x = 011	1	0	0	0
x = 100	0	0	1	0
x = 101	0	0	0	1
x = 110	0	1	0	0
x = 111	0	1	0	0

**Table 5:** Channel $C_{\mathrm{cons},101}$ modeling the case in which the defender runs a constant-time checker and the attacker picks low-input 101.

$C_{\mathrm{cons},101}$	*y* = (*F*, 3)	*y* = (*T*, 3)
x = 000	1	0
x = 001	1	0
x = 010	1	0
x = 011	1	0
x = 100	1	0
x = 101	0	1
x = 110	1	0
x = 111	1	0

**Table 6:** Payoff for each pure strategy profile of 3-bit password scenario.

		Attacker’s Action *a*							
	U(d, a)	000	001	010	011	100	101	110	111
Defender’s action *d*	123	0.7257	0.7257	0.9311	0.9311	0.6577	0.6577	0.7122	0.7122
	132	0.8900	0.9311	0.8900	0.9311	0.7122	0.7122	0.7122	0.7122
	213	0.5068	0.5068	0.9311	0.9311	0.4934	0.4934	0.7668	0.7668
	231	0.5068	0.5068	0.7668	0.9311	0.5068	0.5068	0.7668	0.9311
	312	0.7257	0.9311	0.7257	0.9311	0.7122	0.8766	0.7122	0.8766
	321	0.6712	0.7122	0.7257	0.9311	0.6712	0.7122	0.7257	0.9311

**Table 7:** Payoff table for each pure strategy profile of the 3-bit password scenario, under prior $\pi^{\left(A\right)}$.

		Attacker’s Action *a*							
	U(d, a)	000	001	010	011	100	101	110	111
Defender’s action *d*	123	0.75	0.75	0.75	0.75	0.25	0.25	0.25	0.25
	132	0.75	0.75	0.75	0.75	0.25	0.25	0.25	0.25
	213	0.75	0.75	0.75	0.75	0.50	0.50	0.50	0.50
	231	0.75	0.75	0.75	0.75	0.75	0.75	0.75	0.75
	312	0.75	0.75	0.75	0.75	0.50	0.50	0.50	0.50
	321	0.75	0.75	0.75	0.75	0.75	0.75	0.75	0.75

**Table 8:** Payoff table for each pure strategy profile of the 3-bit password scenario, under prior $\pi^{\left(B\right)}$.

		Attacker’s Action *a*							
	U(d, a)	000	001	010	011	100	101	110	111
Defender’s action *d*	123	0.70	0.70	0.60	0.60	0.50	0.50	0.45	0.4
	132	0.70	0.65	0.70	0.65	0.50	0.50	0.50	0.5
	213	0.70	0.70	0.55	0.55	0.60	0.60	0.50	0.5
	231	0.70	0.70	0.55	0.55	0.70	0.70	0.55	0.5
	312	0.70	0.65	0.70	0.65	0.60	0.60	0.60	0.6
	321	0.70	0.65	0.65	0.60	0.70	0.65	0.65	0.6

## Appendix A. Proofs of Technical Results

In this section, we provide the proofs of the technical results, which are not in the main body of the paper.

### Appendix A.1. Preliminaries for Proofs

We start by providing some necessary background for the subsequent technical proofs.

Definition 1 states that two compatible channels (i.e., with the same input space) are equivalent if yield the same value of vulnerability for every prior and every vulnerability function. The result below, from the literature, provides necessary and sufficient conditions for two channels being equivalent. The result employs the extension of channels with an all-zero column as follows. For any channel *C* of type X×Y→R, its zero-column extension $C^{0}$ is the channel of type $X\times \left(Y\cup \{y_{0}\}\right)\to R$, with $y_{0}\notin Y$, s.t. $C^{0}\left(x,y\right)=C\left(x,y\right)$ for all x∈X, y∈Y, and $C^{0}\left(x,y_{0}\right)=0$ for all x∈X.

**Lemma** **A1** (Characterization of channel equivalence [13,17])**.** *Two channels $C_{1}$ and $C_{2}$ are equivalent iff every column of $C_{1}$ is a convex combination of columns of $C_{2}^{0}$, and every column of $C_{2}$ is a convex combination of columns of $C_{1}^{0}$.*

Note that the result above implies that for being equivalent, any two channels must be compatible.

### Appendix A.2. Proofs of Section 4

**Proposition** **1** (Type of hidden choice)**.** Given a family ${\left\{C_{i}\right\}}_{i\in I}$ of channels of type X×Y→R, and a distribution μ on I, the hidden choice ${⨊}_{i\leftarrow \mu }C_{i}$ is a channel of type X×Y→R.

**Proof.** Since hidden choice is defined as a summation of matrices, the type of ${⨊}_{i\leftarrow \mu }C_{i}$ is the same as the type of every $C_{i}$ in the family. To see that ${⨊}_{i\leftarrow \mu }C_{i}$ is a channel (i.e., all of its entries are non-negative, and all of its rows sum up to 1), first note that, since each $C_{i}$ in the family is a channel matrix, $C_{i}\left(x,y\right)$ lies in the interval [0,1] for all x∈X, y∈Y. Since μ is a set of convex coefficients, from the definition of hidden choice it follows that also $\sum_{i\in I}\mu \left(i\right)C_{i}\left(x,y\right)$ must lie in the interval [0,1] for every x,y. Second, note that for all x∈X:

$$\begin{array}{ccc} \sum_{y\in Y}\left(\underset{i\leftarrow \mu }{⨊}C_{i}\right)\left(x,y\right)= & \;\sum_{y\in Y}\sum_{i\in I}\mu \left(i\right)C_{i}\left(x,y\right) & \left(\mathrm{def}.\mathrm{of}\;\mathrm{hidden}\;\mathrm{choice}\right)\\ = & \;\sum_{i\in I}\mu \left(i\right)\sum_{y\in Y}C_{i}\left(x,y\right)\\ = & \;\sum_{i\in I}\mu \left(i\right)\cdot 1 & \left(C_{i}:X\times Y\to R\;\mathrm{are}\;\mathrm{channels}\right)\\ = & \;1 & \left(\mu \;\mathrm{is}\;a\;\mathrm{prob}.\mathrm{dist}.\right) \end{array}$$

 ☐

**Proposition** **2** (Type of visible choice)**.** *Given a family ${\left\{C_{i}\right\}}_{i\in I}$ of compatible channels s.t. each $C_{i}$ has type $X\times Y_{i}\to R$ and a distribution μ on I, the result of the visible choice ${\left\lfloor \;\cdot \;\right\rfloor }_{i\leftarrow \mu }C_{i}$ is a channel of type $X\times \left({⨆}_{i\in I}Y_{i}\right)\to R$.*

**Proof.** Visible choice applied to a family $\left\{C_{i}\right\}$ of channels scales each matrix $C_{i}$ by a factor μ(i), which preserves the type $X\times Y_{i}\to R$ of each matrix, and then concatenates all the matrices so produced, yielding a result of type $X\times \left({⨆}_{i\in I}Y_{i}\right)\to R$. To see that ${\left\lfloor \;\cdot \;\right\rfloor }_{i\leftarrow \mu }C_{i}$ is a channel (i.e., that all of its entries are non-negative, and that all rows sum-up to 1), note that each element of the visible choice on the family $\left\{C_{i}\right\}$ can be denoted by $\left({\left\lfloor \;\cdot \;\right\rfloor }_{i\leftarrow \mu }C_{i}\right)\left(x,\left(y,j\right)\right)$, where x∈X, j∈I, and $y\in Y_{j}$. Then, note that for all x∈X, j∈I, and $y\in Y_{j}$:

(A1) $$\begin{array}{ccc} \left({\left\lfloor \;\cdot \;\right\rfloor }_{i\leftarrow \mu }C_{i}\right)\left(x,\left(y,j\right)\right)= & \;\left({⋄}_{i\in I}\mu \left(i\right)C_{i}\right)\left(x,\left(y,j\right)\right) & \left(\mathrm{def}.\;\mathrm{of}\;\mathrm{visible}\;\mathrm{choice}\right)\\ = & \;\left(\mu \left(j\right)C_{j}\right)\left(x,y\right) & \left(\mathrm{def}.\;\mathrm{of}\;\mathrm{concatenation}\right)\\ = & \;\mu \left(j\right)C_{j}\left(x,y\right) & \left(\mathrm{def}.\;\mathrm{of}\;\mathrm{scalar}\;\mathrm{mult}.\right) \end{array}$$

which is a non-negative value, since, both μ(j) and $C_{j}\left(x,y\right)$ are non-negative. Finally, note that for all x∈X:

$$\begin{array}{ccc} \sum_{\begin{array}{c} j\in I\\ y\in Y_{j} \end{array}}\left({\left\lfloor \;\cdot \;\right\rfloor }_{i\leftarrow \mu }C_{i}\right)\left(x,\left(y,j\right)\right)= & \;\sum_{\begin{array}{c} j\in I\\ y\in Y_{j} \end{array}}\mu \left(j\right)C_{j}\left(x,y\right) & \left(\mathrm{by}\;\mathrm{Equation}\;(A1)\right)\\ = & \;\sum_{j\in I}\mu \left(j\right)\sum_{y\in Y_{j}}C_{j}\left(x,y\right) & \\ = & \;\sum_{j\in I}\mu \left(j\right)\cdot 1 & \left(C_{j}:X\times Y_{j}\to R\;\mathrm{are}\;\mathrm{channels}\right)\\ = & \;1 & \left(\mu \;\mathrm{is}\;a\;\mathrm{prob}.\;\mathrm{dist}.\right) \end{array}$$

 ☐

**Proposition** **3** (Idempotency)**.** *Given a family ${\left\{C_{i}\right\}}_{i\in I}$ of channels s.t. $C_{i}=C$ for all i∈I, and a distribution μ on I, then: (a) ${⨊}_{i\leftarrow \mu }C_{i}=C$; and (b) ${\left\lfloor \;\cdot \;\right\rfloor }_{i\leftarrow \mu }C_{i}\approx C$.*

**Proof.** 

(a) Idempotency of hidden choice:
  $$\begin{array}{ccc} {⨊}_{i\leftarrow \mu }C_{i}= & \;\sum_{i}\mu \left(i\right)C_{i} & \left(\mathrm{def}.\;\mathrm{of}\;\mathrm{hidden}\;\mathrm{choice}\right)\\ = & \;\sum_{i}\mu \left(i\right)C & \left(\sin\mathrm{ce}\;\mathrm{every}\;C_{i}=C\right)\\ = & \;C\sum_{i}\mu \left(i\right) & \\ = & \;C & \left(\sin\mathrm{ce}\;\mu \;\mathrm{is}\;a\;\mathrm{prob}.\;\mathrm{dist}.\right) \end{array}$$
(b) Idempotency of visible choice:
  $$\begin{array}{ccc} {\left\lfloor \;\cdot \;\right\rfloor }_{i\leftarrow \mu }C_{i}= & \;{⋄}_{i}\mu \left(i\right)C_{i} & \left(\mathrm{def}.\;\mathrm{of}\;\mathrm{visible}\;\mathrm{choice}\right)\\ = & \;{⋄}_{i}\mu \left(i\right)C & \left(\sin\mathrm{ce}\;\mathrm{every}\;C_{i}=C\right)\\ \approx & \;C & \left(\mathrm{by}\;\mathrm{Lemma}\;A1\right) \end{array}$$
  In the above derivation, we can apply Lemma A1 because every column of the channel on each side of the equivalence can be written as a convex combination of the zero-column extension of the channel on the other side.

 ☐

**Proposition** **4** (“Reorganization of operators”)**.** *Given a family ${\left\{C_{ij}\right\}}_{i\in I,j\in J}$ of channels indexed by sets I and J, a distribution μ on I and a distribution η on J*:

(a) ${⨊}_{i\leftarrow \mu }\;{⨊}_{j\leftarrow \eta }C_{ij}={⨊}_{\begin{array}{c} i\leftarrow \mu \\ j\leftarrow \eta \end{array}}C_{ij}$, if all $C_{i}$’s have the same type;
(b) ${\left\lfloor \;\cdot \;\right\rfloor }_{i\leftarrow \mu }\;{\left\lfloor \;\cdot \;\right\rfloor }_{j\leftarrow \eta }C_{ij}\approx {\left\lfloor \;\cdot \;\right\rfloor }_{\begin{array}{c} i\leftarrow \mu \\ j\leftarrow \eta \end{array}}C_{ij}$, if all $C_{i}$’s are compatible; and
(c) ${⨊}_{i\leftarrow \mu }\;{\left\lfloor \;\cdot \;\right\rfloor }_{j\leftarrow \eta }C_{ij}\approx {\left\lfloor \;\cdot \;\right\rfloor }_{j\leftarrow \eta }\;{⨊}_{i\leftarrow \mu }C_{ij}$, if, for each i, all $C_{ij}$’s have the same type $X\times Y_{j}\to R$.

**Proof.** 

(a) $$\begin{array}{ccc} \underset{i\leftarrow \mu }{⨊}\underset{j\leftarrow \eta }{⨊}C_{ij}= & \;\underset{i\leftarrow \mu }{⨊}\left(\sum_{j}\eta \left(j\right)C_{ij}\right) & \left(\mathrm{def}.\;\mathrm{of}\;\mathrm{hidden}\;\mathrm{choice}\right)\\ = & \;\sum_{i}\mu \left(i\right)\left(\sum_{j}\eta \left(j\right)C_{ij}\right) & \left(\mathrm{def}.\;\mathrm{of}\;\mathrm{hidden}\;\mathrm{choice}\right)\\ = & \;\sum_{i,j}\eta \left(i\right)\eta \left(j\right)C_{ij} & \left(\mathrm{reorganizing}\;\mathrm{the}\;\mathrm{sums}\right)\\ = & \;\underset{\begin{array}{c} i\leftarrow \mu \\ j\leftarrow \eta \end{array}}{⨊}C_{ij} & \left(\mathrm{def}.\;\mathrm{of}\;\mathrm{hidden}\;\mathrm{choice}\right) \end{array}$$
(b) $$\begin{array}{ccc} \underset{i\leftarrow \mu }{\left\lfloor \;\cdot \;\right\rfloor }\underset{j\leftarrow \eta }{\left\lfloor \;\cdot \;\right\rfloor }C_{ij}= & \;\underset{i\leftarrow \mu }{\left\lfloor \;\cdot \;\right\rfloor }\left({⋄}_{j}C_{ij}\right) & \left(\mathrm{def}.\;\mathrm{of}\;\mathrm{visible}\;\mathrm{choice}\right)\\ = & \;{⋄}_{i}\mu \left(i\right)\left({⋄}_{j}\eta \left(j\right)C_{ij}\right) & \left(\mathrm{def}.\;\mathrm{of}\;\mathrm{visible}\;\mathrm{choice}\right)\\ \approx & {⋄}_{ij}\mu \left(i\right)\eta \left(j\right)C_{ij} & \left(\mathrm{by}\;\mathrm{Lemma}\;A1\right)\\ = & \;\underset{\begin{array}{c} i\leftarrow \mu \\ j\leftarrow \eta \end{array}}{\left\lfloor \;\cdot \;\right\rfloor }C_{ij} & \left(\mathrm{def}.\;\mathrm{of}\;\mathrm{visible}\;\mathrm{choice}\right) \end{array}$$
  In the above derivation, we can apply Lemma A1 because every column of the channel on each side of the equivalence can be written as a convex combination of the zero-column extension of the channel on the other side.
(c) $$\begin{array}{ccc} \underset{i\leftarrow \mu }{⨊}\underset{j\leftarrow \eta }{\left\lfloor \;\cdot \;\right\rfloor }C_{ij}= & \;\underset{i\leftarrow \mu }{⨊}\left({⋄}_{j}C_{ij}\right) & \left(\mathrm{def}.\;\mathrm{of}\;\mathrm{visible}\;\mathrm{choice}\right)\\ = & \;\sum_{i}\mu \left(i\right)\left({⋄}_{j}\eta \left(j\right)C_{ij}\right) & \left(\mathrm{def}.\;\mathrm{of}\;\mathrm{hidden}\;\mathrm{choice}\right)\\ \approx & \;{⋄}_{j}\eta \left(j\right)\left(\sum_{i}\eta \left(i\right)C_{ij}\right) & \left(\mathrm{by}\;\mathrm{Lemma}\;A1\right)\\ = & \;\underset{j\leftarrow \eta }{\left\lfloor \;\cdot \;\right\rfloor }\underset{i\leftarrow \mu }{⨊}C_{ij} & \left(\mathrm{def}.\;\mathrm{of}\;\mathrm{operators}\right) \end{array}$$
  In the above derivation, we can apply Lemma A1 because every column of the channel on each side of the equivalence can be written as a convex combination of the zero-column extension of the channel on the other side.

 ☐

### Appendix A.3. Proofs of Section 7

**Theorem** **A1****.** Consider the password checker program of Algorithm 1 for *n*-bit passwords, where the attacker controls the low input to the checker and the defender controls the order in which the bits are checked. If the prior π on possible passwords is uniform and the payoff is given by the posterior Bayes-vulnerability: $U\left(\delta ,\alpha \right)=E_{\begin{array}{c} a\leftarrow \alpha \end{array}}\;V\left[\pi ,{⨊}_{d\leftarrow \delta }C_{da}\right]$, then the strategy $\left(\delta^{*},\alpha \right)$ where $\delta^{*}$ is uniform and α is arbitrary is an equilibrium strategy.

**Proof.** We show that $\left(\delta^{*},\alpha \right)$ where $\delta^{*}$ is uniform and α is arbitrary, is a saddle point of U(δ,α). The proof will rely on two crucial symmetries of the password checker, in combination with the use of a uniform prior. Note that, under uniform π, the Bayes-vulnerability of a channel *C* is proportional to the sum of the column maxima of *C*, namely $V\left[\pi ,C\right]=\frac{1}{\left|X\right|}\sum_{y}\max_{x}C\left(x,y\right)$. Given a permutation σ of X, define $C^{\sigma }$ as *C* with its rows permuted, namely $C^{\sigma }\left(x,y\right)=C\left(\sigma \left(x\right),y\right)$. Note that $C^{\sigma }$ can be written as $M_{\sigma }C$ where $M_{\sigma }$ is a permutation matrix. Permuting the rows does not affect the column maxima, hence $V\left[\pi ,C\right]=V\left[\pi ,C^{\sigma }\right]$. For the attacker things are simple, since we can show that under a uniform π all attacker strategies are equivalent, that is,

$$U\left(\alpha ,\delta \right)=U\left(\alpha^{\prime },\delta \right)\;\mathrm{for}\;\mathrm{all}\alpha ,\alpha^{\prime },\delta \;.$$

To show this, we use the first important symmetry of the password checker, which is due to the fact that the output of the algorithm only depends on whether $x_{i}\neq a_{i}$ is true or false for each bit. Hence, any modification on *a* can be matched with a modification on *x* that preserves the output, namely for all $a,a^{\prime }$ there exists a permutation σ of passwords such that $C_{da}=C_{da^{\prime }}^{\sigma }$ for all *d*. Denote $C_{\delta a}={⨊}_{d\leftarrow \delta }C_{da}$, we have that:

$$C_{\delta a}=\underset{d\leftarrow \delta }{⨊}C_{da}=\underset{d\leftarrow \delta }{⨊}M_{\sigma }C_{da^{\prime }}=M_{\sigma }\underset{d\leftarrow \delta }{⨊}C_{da^{\prime }}=C_{\delta a^{\prime }}^{\sigma }$$

From $V\left[\pi ,C_{\delta a}\right]=V\left[\pi ,C_{\delta a^{\prime }}^{\sigma }\right]$, we have that $U\left(a,\delta \right)=U\left(a^{\prime },\delta \right)$ for all pure strategies $a,a^{\prime }$, which implies $U\left(\alpha ,\delta \right)=U\left(\alpha^{\prime },\delta \right)$ since U(α,δ) is linear on α. Therefore, in the remainder of the proof, we assume that the attacker plays the fixed pure strategy $a_{0}$, having the zero bitstring 0…0 as the low input. For the defender, on the other hand, the situation is far from trivial: although all pure strategies *d* are still equivalent, U(α,δ) is only convex on δ and as a consequence the payoff highly depends on δ. Our goal is to show that:

$$U\left(a_{0},\delta \right)=\frac{1}{\left|X\right|}\sum_{y}\max_{x}\sum_{d}\delta \left(d\right)C_{da_{0}}\left(x,y\right)$$

is minimized on the uniform $\delta^{*}$. To do so, we show something stronger, namely that each addend of the *y*-sum is simultaneously minimized on the uniform $\delta^{*}$. Fix an arbitrary *y*, and let:

$$f\left(\delta \right)=\max_{x}\delta \cdot \psi_{x}$$

where · is the dot product and $\psi_{x}\in R^{\left|D\right|}$ is the vector defined by $\psi_{x}\left(d\right)=C_{da_{0}}\left(x,y\right)$. Note that, since $C_{da_{0}}$ is deterministic, all elements of $\psi_{x}$ are either zero or one. Intuitively, $\psi_{x}\left(d\right)=1$ if *x* produces the fixed output *y* when the bit checking order is *d*. We need to show that f(δ) is minimized on $\delta^{*}$. However, *f* seen as a function on the whole $R^{\left|D\right|}$ has no global minimum. In our case, though, δ is a probability distribution taking values in $DD\subset R^{\left|D\right|}$. That is, only |D|−1 elements of δ are free, so we can reduce the dimension by one as follows: fix some $d_{0}\in D$ and let $\tilde{\delta },{\tilde{\psi }}_{x}\in R^{|D|-1}$ be the same as $\delta ,\psi_{x}$ with the element corresponding to $d_{0}$ removed. We have that $\delta \left(d_{0}\right)=1-\sum_{d\neq d_{0}}\delta \left(d\right)=1-\tilde{\delta }\cdot 1$, where 1 is the “ones” vector; hence, we can rewrite f(δ) as a function of $\tilde{\delta }$:

$$\begin{array}{cc} f(\tilde{\delta }) & =\max_{x}\tilde{\delta }\cdot {\tilde{\psi }}_{x}+\delta \left(d_{0}\right)\psi_{x}\left(d_{0}\right)\\ & =\max_{x}\tilde{\delta }\cdot {\tilde{\psi }}_{x}+\left(1-\tilde{\delta }\cdot 1\right)\psi_{x}\left(d_{0}\right)\\ & =\max_{x}\tilde{\delta }\cdot \left({\tilde{\psi }}_{x}-\psi_{x}\left(d_{0}\right)1\right)+\psi_{x}\left(d_{0}\right) \end{array}$$

Therefore, it is sufficient to show that $f(\tilde{\delta })$ is minimized on ${\tilde{\delta }}^{*}$. In the following, we use the fundamental concept of subgradients from convex analysis, which generalize gradients for non-differentiable functions. A vector *v* is a subgradient of a possibly non-differentiable convex function g:S→R at $x_{0}\in S$ iff:

$$g\left(x\right)-g\left(x_{0}\right)\geq v\cdot \left(x-x_{0}\right)\;\mathrm{for}\;\mathrm{all}\;x\in S.$$

It is well-known that *g* has a global minimum on $x_{0}$ iff the 0 vector belongs to the set of subgradients of *g* at $x_{0}$ (this generalizes the fact that differentiable convex functions have zero gradient on their global minimum). Recall also that g(x)=x·v+c has a single subgradient *v*, while for $g\left(x\right)=\max_{i}g_{i}\left(x\right)$, any subgradient of $g_{i}$ for any branch *i* giving the max, is also a subgradient of *g*. Finally, the set of subgradients is convex, so any convex combination of them is also a subgradient. Hence, the subgradients of $f(\tilde{\delta })$ are ${\tilde{\psi }}_{x}-\psi_{x}\left(d_{0}\right)1$, for any *x* giving the maximum, as well as their convex combinations. Our goal is to show that on ${\tilde{\delta }}^{*}$ these include the zero vector. We finally arrive to the second important symmetry of the password checker. Let ρ be a permutation of the set {1,…,n} of password bits. Such a permutation could be seen both as a permutation on X (i.e., $\rho \left(x\right)=x_{\rho (1)}\ldots x_{\rho (n)}$; note that ρ(x) has the same number of 0s and 1s as *x*), as well as a permutation on D (a bit checking order *d* is itself a permutation of bits, so we can set ρ(d)=ρ∘d). Since all low bits of $a_{0}$ are the same, applying ρ to both *x* and *d* does not change the outcome of the algorithm, that is $C_{da_{0}}=C_{\rho \left(d\right)a_{0}}^{\rho }$. Intuitively, if *d* is selected uniformly, then the attacker cannot distinguish *x* from ρ(x) since they produce the same output with the same probability. More concretely, let $x∼x^{\prime }$ denote that $x^{\prime }=\rho \left(x\right)$ for some bit permutation ρ. Due to the aforementioned symmetry we have that $\psi_{x}$ and $\psi_{x^{\prime }}$ have the same number of 1s which means that $\delta^{*}\cdot \psi_{x}=\delta^{*}\cdot \psi_{x^{\prime }}$. Now, let $x^{*}\in {\mathrm{argmax}}_{x}\delta^{*}\cdot \psi_{x}$. We have that all $x∼x^{*}$ also give the max; hence, all vectors:

(A2) $${\tilde{\psi }}_{x}-\psi_{x}\left(d_{0}\right)1\;x∼x^{*}$$

are subgradients of *f* on ${\tilde{\delta }}^{*}$. Finally, for any $d,d^{\prime }$ there is a bit permutation ρ such that $d^{\prime }=\rho \left(d\right)$. Since $\psi_{x}\left(d\right)=\psi_{\rho (x)}\left(\rho \left(d\right)\right)$, we have that $\left(\sum_{x∼x^{*}}\psi_{x}\right)\left(d\right)=k$ (for some integer *k*) independently of *d*. Hence, letting $c=|\{x\;|\;x∼x^{*}\}|$ and averaging all subgradients of (A2), we get that:

$$\frac{1}{c}\sum_{x∼x^{*}}\left({\tilde{\psi }}_{x}-\psi_{x}\left(d_{0}\right)1\right)=\frac{1}{c}\left(k1-k1\right)=0$$

is also a subgradient, which concludes the proof. ☐

## Appendix B. Properties of Binary Versions of Channel Operators

In this section, we derive some relevant properties of the binary versions of the hidden and visible choice operators. We start with results regarding each operator individually.

**Proposition** **A1** (Properties of the binary hidden choice)**.** *For any channels $C_{1}$ and $C_{2}$ of the same type, and any values 0≤p,q≤1, the binary hidden choice operator satisfies the following properties:*

(a) Idempotency: $C_{1}\;{}_{p}⊕\;C_{1}=C_{1}$
(b) Commutativity: $C_{1}\;{}_{p}⊕\;C_{2}=C_{2}\;{}_{\bar{p}}⊕\;C_{1}$
(c) *Associativity:*
  C1p⊕(C2q⊕C3)=(1q·C1p⊕C2)q⊕p¯·C3
  *if q≠0.*
(d) *Absorption:*
  $$\left(C_{1}\;{}_{p}⊕\;C_{2}\right)\;{}_{q}⊕\;\left(C_{1}\;{}_{r}⊕\;C_{2}\right)=C_{1}\;{}_{\left(pq+\bar{q}r\right)}⊕\;C_{2}.$$

**Proof.** We will prove each property separately.

(a) Idempotency:
  $$\begin{array}{ccc} C_{1}\;{}_{p}⊕\;C_{1}= & \;p\cdot C_{1}+\bar{p}\cdot C_{1} & \left(\mathrm{def}.\;\mathrm{of}\;\mathrm{hidden}\;\mathrm{choice}\right)\\ = & \;\left(p+\bar{p}\right)\cdot C_{1} & \\ = & \;C_{1} & \left(p+\bar{p}=1\right) \end{array}$$
(b) Commutativity:
  $$\begin{array}{ccc} C_{1}\;{}_{p}⊕\;C_{2}= & \;p\cdot C_{1}+\bar{p}\cdot C_{2} & \left(\mathrm{def}.\;\mathrm{of}\;\mathrm{hidden}\;\mathrm{choice}\right)\\ = & \;\bar{p}\cdot C_{2}+p\cdot C_{1} & \\ = & \;C_{2}\;{}_{\bar{p}}⊕\;C_{1} & \left(\mathrm{def}.\;\mathrm{of}\;\mathrm{hidden}\;\mathrm{choice}\right) \end{array}$$
(c) Associativity:
  C1p⊕(C2q⊕C3)=p·C1+p¯(q·C2+q¯·C3)(def.ofhiddenchoice)=p·C1+p¯q·C2+p¯q¯·C3=q(p(1q·C1)+p¯·C2)+q¯(p¯·C3)=(1q·C1p⊕C2)q⊕p¯·C3(def.ofhiddenchoice)
(d) Absorption: First note that:
  $$\begin{array}{ccc} & \;\left(C_{1}\;{}_{p}⊕\;C_{2}\right)\;{}_{q}⊕\;\left(C_{1}\;{}_{r}⊕\;C_{2}\right) & \\ = & \;q\left(pC_{1}+\bar{p}C_{2}\right)+\bar{q}\left(rC_{1}+\bar{r}C_{2}\right) & \left(\mathrm{def}.\;\mathrm{of}\;\mathrm{hidden}\;\mathrm{choice}\right)\\ = & \;pqC_{1}+\bar{p}qC_{2}+\bar{q}rC_{1}+\bar{q}\bar{r}C_{2} & \\ = & \;\left(pq+\bar{q}r\right)C_{1}+\left(\bar{p}q+\bar{q}\bar{r}\right)C_{2}\\ = & \;C_{1}\;{}_{\left(pq+\bar{q}r\right)}⊕\;C_{2} & \left(*\right) \end{array}$$
  To complete the proof, note that in step (*) above, $pq+\bar{q}r$ and $\bar{p}q+\bar{q}\bar{r}$ form a valid binary probability distribution (they are both non-negative, and they add up to one), then apply the definition of hidden choice.

 ☐

**Proposition** **A2** (Properties of binary visible choice)**.** *For any compatible channels $C_{1}$ and $C_{2}$, and any values 0≤p,q≤1, the visible choice operator satisfies the following properties:*

(a) Idempotency: $C_{1}\;{}_{p}\left\lfloor \;\cdot \;\right\rfloor \;C_{1}\approx C_{1}$
(b) Commutativity: $C_{1}\;{}_{p}\left\lfloor \;\cdot \;\right\rfloor \;C_{2}\approx C_{2}\;{}_{\bar{p}}\left\lfloor \;\cdot \;\right\rfloor \;C_{1}$
(c) Associativity: C1p⌊·⌋(C2q⌊·⌋C3)≈(1q·C1p⌊·⌋C2)q⌊·⌋p¯·C3 if q≠0.

**Proof.** We will prove each property separately.

(a) Idempotency: $C_{1}\;{}_{p}\left\lfloor \;\cdot \;\right\rfloor \;C_{1}\approx C_{1}$, by immediate application of Lemma A1, since every column of the channel on each side of the equivalence can be written as a convex combination of the zero-column extension of the channel on the other side.
(b) Commutativity:
  $$\begin{array}{ccc} C_{1}\;{}_{p}\left\lfloor \;\cdot \;\right\rfloor \;C_{2}= & \;p\cdot C_{1}⋄\bar{p}\cdot C_{2} & \left(\mathrm{def}.\;\mathrm{of}\;\mathrm{visible}\;\mathrm{choice}\right)\\ \approx & \;\bar{p}\cdot C_{2}⋄p\cdot C_{1} & \left(\mathrm{by}\;\mathrm{Lemma}\;A1\right)\\ = & \;C_{2}\;{}_{\bar{p}}\left\lfloor \;\cdot \;\right\rfloor \;C_{1} & (\mathrm{def}.\;\mathrm{of}\;\mathrm{visible}\;\mathrm{choice}). \end{array}$$
  In the above derivation, we can apply Lemma A1 because every column of the channel on each side of the equivalence can be written as a convex combination of the zero-column extension of the channel on the other side.
(c) Associativity:
  C1p⌊·⌋(C2q⌊·⌋C3)=p·C1⋄p¯(q·C2⋄q¯·C3)(def.ofvisiblechoice)≈p·C1⋄p¯q·C2⋄p¯q¯·C3(byLemmaA1)=q(p(1q·C1)⋄p¯·C2)⋄q¯(p¯·C3)=(1q·C1p⌊·⌋C2)q⌊·⌋p¯·C3(def.ofvisiblechoice)
  In the above derivation, we can apply Lemma A1 because every column of the channel on each side of the equivalence can be written as a convex combination of the zero-column extension of the channel on the other side.

 ☐

Now, we turn our attention to the interaction between hidden and visible choice operators.

A first result is that hidden choice does not distribute over visible choice. To see why, note that $C_{1}\;{}_{p}⊕\;\left(C_{2}\;{}_{q}\left\lfloor \;\cdot \;\right\rfloor \;C_{3}\right)$ and $\left(C_{1}\;{}_{p}⊕\;C_{2}\right)\;{}_{q}\left\lfloor \;\cdot \;\right\rfloor \;\left(C_{1}\;{}_{p}⊕\;C_{3}\right)$ cannot be both defined: if the former is defined, then $C_{1}$ must have the same type as $C_{2}\;{}_{q}\left\lfloor \;\cdot \;\right\rfloor \;C_{3}$, whereas if the latter is defined, $C_{1}$ must have the same type as $C_{2}$, but $C_{2}\;{}_{q}\left\lfloor \;\cdot \;\right\rfloor \;C_{3}$ and $C_{2}$ do not have the same type (they have different output sets).

However, as the next result shows, visible choice distributes over hidden choice.

**Proposition** **A3** (Distributivity of _*p*_⌊·⌋ over _*q*_⊕)**.** *Let $C_{1}$, $C_{2}$ and $C_{3}$ be compatible channels, and $C_{2}$ and $C_{3}$ have the same type. Then, for any values 0≤p,q≤1:*

$$\begin{array}{c} C_{1}\;{}_{p}\left\lfloor \;\cdot \;\right\rfloor \;\left(C_{2}\;{}_{q}⊕\;C_{3}\right)\approx \left(C_{1}\;{}_{p}\left\lfloor \;\cdot \;\right\rfloor \;C_{2}\right)\;{}_{q}⊕\;\left(C_{1}\;{}_{p}\left\lfloor \;\cdot \;\right\rfloor \;C_{3}\right). \end{array}$$

**Proof.** 

$$\begin{array}{ccc} & \;C_{1}\;{}_{p}\left\lfloor \;\cdot \;\right\rfloor \;\left(C_{2}\;{}_{q}⊕\;C_{3}\right) & \\ = & \;\left(C_{1}\;{}_{q}⊕\;C_{1}\right)\;{}_{p}\left\lfloor \;\cdot \;\right\rfloor \;\left(C_{2}\;{}_{q}⊕\;C_{3}\right) & \left(\mathrm{idempotency}\;\mathrm{of}\;\mathrm{visible}\;\mathrm{choice}\right)\\ = & \;p\left(q\cdot C_{1}+\bar{q}\cdot C_{1}\right)⋄\bar{p}\left(q\cdot C_{2}+\bar{q}\cdot C_{3}\right) & \left(\mathrm{def}.\;\mathrm{of}\;\mathrm{operators}\right)\\ = & \;\left(pq\cdot C_{1}+p\bar{q}\cdot C_{1}\right)⋄\left(\bar{p}q\cdot C_{2}+\bar{p}\bar{q}\cdot C_{3}\right) & \\ \approx & \;\left(pq\cdot C_{1}⋄\bar{p}q\cdot C_{2}\right)+\left(p\bar{q}\cdot C_{1}⋄\bar{p}\bar{q}\cdot C_{3}\right) & \left(\mathrm{by}\;\mathrm{Lemma}\;A1\right)\\ = & \;q\left(p\cdot C_{1}⋄\bar{p}\cdot C_{2}\right)+\bar{q}\left(p\cdot C_{1}⋄\bar{p}\cdot C_{3}\right) & \\ = & \;q\left(C_{1}\;{}_{p}\left\lfloor \;\cdot \;\right\rfloor \;C_{2}\right)+\bar{q}\left(C_{1}\;{}_{p}\left\lfloor \;\cdot \;\right\rfloor \;C_{3}\right) & \left(\mathrm{def}.\;\mathrm{of}\;\mathrm{visible}\;\mathrm{choice}\right)\\ = & \;\left(C_{1}\;{}_{p}\left\lfloor \;\cdot \;\right\rfloor \;C_{2}\right)\;{}_{q}⊕\;\left(C_{1}\;{}_{p}\left\lfloor \;\cdot \;\right\rfloor \;C_{3}\right) & \left(\mathrm{def}.\;\mathrm{of}\;\mathrm{hidden}\;\mathrm{choice}\right) \end{array}$$

In the above derivation, we can apply Lemma A1 because every column of the channel on each side of the equivalence can be written as a convex combination of the zero-column extension of the channel on the other side. ☐
